# Sixteen Years of *Bt* Maize in the EU Hotspot: Why Has Resistance Not Evolved?

**DOI:** 10.1371/journal.pone.0154200

**Published:** 2016-05-04

**Authors:** Pedro Castañera, Gema P. Farinós, Félix Ortego, David A. Andow

**Affiliations:** 1 Department of Environmental Biology, Centro de Investigaciones Biológicas, CSIC, Ramiro de Maeztu 9, 28040 Madrid, Spain; 2 Department of Entomology, University of Minnesota, 219 Hodson Hall, 1980 Folwell Ave., Saint Paul, Minnesota 55108, United States of America; University of Tennessee, UNITED STATES

## Abstract

The majority of *Bt* maize production in the European Union (EU) is concentrated in northeast Spain, which is Europe’s only hotspot where resistance might evolve, and the main target pest, *Sesamia nonagrioides*, has been exposed to Cry1Ab maize continuously since 1998. The cropping system in northeast Spain has some similar characteristics to those that probably led to rapid resistance failures in two other target noctuid maize pests. These include repeated cultivation of *Bt* maize in the same fields, low use of refuges, recurring exposure of larvae to non-high dose concentrations of Cry1Ab toxin during the first years of cultivation, low migratory potential, and production concentrated in an irrigated region with few alternative hosts. Available data reveal no evidence of resistance in *S*. *nonagrioides* after 16 years of use. We explore the possible reasons for this resistance management success using evolutionary models to consider factors expected to accelerate resistance, and those expected to delay resistance. Low initial adoption rates and the EU policy decision to replace Event 176 with MON 810 Bt maize were key to delaying resistance evolution. Model results suggest that if refuge compliance continues at the present 90%, *Bt* maize might be used sustainably in northeast Spain for at least 20 more years before resistance might occur. However, obtaining good estimates of the present *R* allele frequency and level of local assortative mating are crucial to reduce uncertainty about the future success of resistance management.

## Introduction

**Fig 1:**
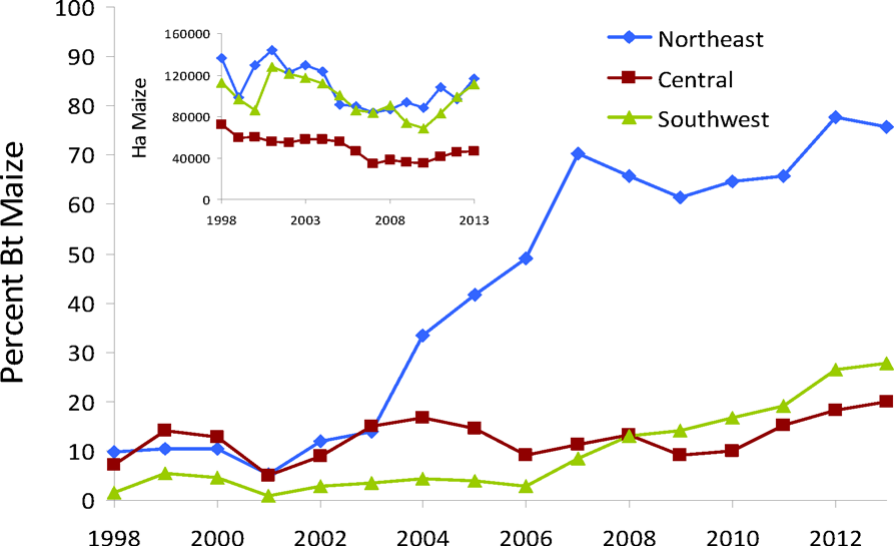
Adoption rate of Bt maize in the three main maize growing areas in Spain. Percentage of Bt maize in northeast (Cataluña and Aragón), central (Madrid and Castilla-La Mancha) and southwest (Extremadura and Andalucía) Spain with respect to the total cultivated maize in each region (inner graph).

The approval in 1998 of Cry1Ab-expressing maize hybrids (*Bt* maize) in the European Union (EU) continues to be debated strongly today. Spain is the only EU country where *Bt* maize has been grown continuously at a large scale, accounting for 92% of the total EU area [1], with most of this in Ebro Valley in northeast Spain, to control recurring damage by the target pest, Mediterranean corn borer, *Sesamia nonagrioides* Lef. [Lepidoptera: Noctuidae] [2]. Adoption of *Bt* maize in northeast Spain has reached >75% (Fig 1), which makes this Europe’s only hotspot, as defined by the European Food Safety Authority (EFSA) [3], where resistance might be expected.

Resistance in the target pests is the major threat to the long-term sustainability of insect resistant transgenic plants. Field-evolved resistance to *Bt* crops has been reported in only five insect pests during the last 17 years [4]. For *Bt* maize, resistance has been documented in two species of noctuid Lepidoptera: African stem borer, *Busseola fusca*, to *Bt* maize expressing Cry1Ab in South Africa [5, 6] and fall armyworm, *Spodoptera frugiperda*, to Cry1F-expressing maize in Puerto Rico and Brazil [7, 8, 9], which occurred after only 8 and 3 years of cultivation, respectively. Resistance can be ascribed to several causes, but factors common to South Africa and Brazil [5, 6, 8, 10, 11] were a) the high adoption rate and low refuge use, b) repeated cultivation of *Bt* maize in the same fields, c) with recurring exposure of larvae of the second and third generation on the crop to non-high dose levels of toxin due to reduced expression of the Bt toxin, d) low migratory potential, and e) production concentrated in an irrigated region where there were few wild or cultivated alternative hosts. In addition, for *B*. *fusca* in the Vaalharts area, there was increased selection pressure due to its preference for irrigated *Bt* maize rather than for adjacent non-irrigated non-*Bt* fields. For *S*. *frugiperda*, its preference for non-damaged maize [12] might have increased selection pressure as it might have preferentially oviposited on undamaged *Bt* maize instead of damaged non-*Bt* maize.

Some of these same factors are common to *S*. *nonagrioides* populations in northeast Spain, where nearly all maize is irrigated and concentrated in a small area and crop rotation is limited. Refuge compliance was low during the first eight years (<60% before 2006) of use of Cry1Ab-expressing *Bt* maize (see Table H in S1 File). Compa CB (Event 176) was the only variety grown until 2003, when it was gradually replaced by MON 810 hybrids and disallowed in 2006. Whereas MON 810 varieties express the Cry1Ab toxin in vegetative and reproductive stages at similar levels [13], the toxin titer for Compa CB decreased as the crop reached maturation [14], exposing larvae of the second and third generation *S*. *nonagrioides* to non-high dose concentrations of Cry1Ab toxin [15, 16], which would accelerate resistance by reducing selection against heterozygotes. Similarly, long distance migration in this species may be rare [17], which allows local selection to dominate the evolution of resistance. In addition, *S*. *nonagrioides* is functionally monophagous on maize in the region. Thus, there are some factors that would suggest that the resistance risk for *S*. *nonagrioides* could be high. At the same time, there are other factors that would suggest a reduced resistance risk for this species, including low initial adoption of Bt maize, and elimination of Event 176. See S1 File (Supporting Information) for more details on the biology of *S*. *nonagrioides* and maize cultivation in northeast Spain (Figs A-C in S1 File).

Here we establish that resistance has not been detected in *S*. *nonagrioides* in northeast Spain after 16 years of *Bt* maize cultivation, by measuring susceptibility in this hotspot and two other locations with historically low levels of adoption of *Bt* maize (Fig 1) and by comparing with information from EFSA [18]. Next we investigate possible reasons why resistance not been detected despite high exposure to a single *Bt* maize toxin. We considered factors expected to accelerate resistance (e.g., low refuge compliance, low female movement, and high local positive assortative mating), and those expected to delay resistance (e.g., low adoption, external colonization, and replacement of Event 176) using a resistance evolution model, parameterized for *S*. *nonagrioides*.

## Materials and Methods

No specific permissions were required at any of the study locations for all of our activities. In Spain, permission for collections is not required because the species is a maize pest. Furthermore, the field sampling was part of the Spanish Post-Market Resistance Monitoring Programme for *Bt*-maize cultivation, which is required and coordinated by the Spanish Ministerio de Agricultura, Alimentación y Medio Ambiente (MAAMA). As a part of their responsibility, the MAAMA also informed farmers that samples were taken and explained the purpose of the study. This study did not involve endangered or protected species.

### Bioassays

A unique batch of Cry1Ab crystals (81% purity), isolated from a bacterial culture of *B*. *thuringiensis* ssp. *kurstaki* HD1-9 strain and provided by Syngenta in 1999 [16], was used for all the bioassays. The Cry1Ab crystals were stored at -20°C and every year a toxin stock solution was prepared by suspending the crystals in a 0.1% solution of Triton X-100 to perform the bioassays programmed for that season.

Last instar pre-diapausing larvae of *S*. *nonagrioide*s from the final generation of the season were collected from maize fields from northeast, central and southwest Spain (2–4 sampling sites from each geographical area, between 100–200 larvae from each site making a total of about 500 larvae, see S2 File for the geographic coordinates for the sampling sites) and the susceptibility to Cry1Ab was tested on the F1 progeny. In addition, a laboratory colony established in 1998, before Bt maize cultivation, and refreshed every two years with individuals collected from non-*Bt* fields (CIB, Madrid, Spain) was used as control. Bioassays were performed in “Bio-Ba-128” plastic trays (Color-Dec Italy, Capezzano Pianore, Italy) containing 128 wells, where ≈ 0.5 ml of rearing diet [16] were placed, flattened and let solidify. Serial dilutions (7 to 10 different concentrations) of Cry1Ab toxin that elicited insect responses ranging from near absent to near complete mortality, excluding dilutions with 0% and 100% responses, plus a control were tested. Fifty μl of each concentration was added in each well on the surface of the diet (1.767 cm^2^). The controls consisted of the buffer solution used to dilute the toxin. The treated trays were let dry in a laminar flow hood and shortly afterwards one neonate larva (<24 h old) was placed in each well and confined with a cover (Bio-Cv-16, Color-Dec Italy, Capezzano Pianore, Italy). Three replicates of 16–32 larvae at each concentration and 32–64 larvae for the control were performed. Neonates from different oviposition cages were used in each replicate. Trays were incubated in growth chambers at 25 ± 1°C, 70 ± 5% r.h. and constant dark. Larval mortality (larvae that did not show any reaction when prodded) was recorded after 7 days. The maximum mortality allowed in controls was 20%. Lethal concentrations (LC_50_ and LC_90_) values with a 95% confidence interval were estimated by probit analysis using POLO-PC (LeOra Software). Resistance Ratios (RR) with 95% confidence limits were calculated with respect to the LC_50_ or MIC_50_ (molt inhibition concentration) values obtained for the susceptible laboratory strain each year to normalize the data [19].

### Mathematical Model

The resistance evolution model is based on Comins (1977) [20,21,22, 23], and is a single-locus, two-allele, deterministic, three-patch, discrete generation model with stochastic weather that determines the diapause rate and survival of the third generation (Fig D in S1 File). Each generation is modelled by tracking population density and allele frequencies through adult emergence, pre-mating movement, mating, post-mating movement, reproduction, selection, density-dependent larval survival, and adult emergence. Each generation has a unique parameterization based on the ecology of *S*. *nonagrioides*. The full model is a system of six coupled non-linear difference equations characterizing resistance allele frequency and population size in three different habitat patches (see SI for mathematical details). The three habitat patches are Mon810 Bt maize, Event 176 Bt maize, and non-Bt maize. For each generation there are 24 parameters, and most of the parameters are allowed to vary over the three generations per year. Nearly all of the refuge plants in northeast Spain are non-*Bt* maize. Resistance is assumed to be conferred by a single resistance allele that gives resistance to both Mon810 and Event 176. Selection occurs on larvae prior to density-dependent mortality. Adult moths disperse and mate. Dispersal is allowed to vary among males, virgin females and mated females. Mating can be locally assortative or disassortative.

### Parameter Estimation

All parameters were estimated independently, based on the best information available on the ecology and genetics of *S*. *nonagrioides*, and the maize cropping system in northeast Spain, and the set of parameter values is referred to as the Best Parameter Values (BPV). For example, the proportion of non-Bt maize refuge is taken from historical records of planting of *Bt* maize in northeast Spain (Fig 1). Future projections assume that adoption of *Bt* maize rises up to the limit allowed by the refuge policy in Spain and the European Union (80% of the area planted to maize in northeast Spain). The initial resistance allele frequency in *S*. *nonagrioides* was estimated by using an F_2_ screen and a Bayesian estimator [24], resulting in a best available estimate of initial resistance allele frequency of 1.5 x 10^−3^. Selection will be different in Event 176 and MON 810, because toxin concentration in Event 176 declines after maize anthesis [14]. Selection parameters are equivalent to a high dose for the first generation on Event 176 and for all generations on Mon810, and heterozygosity increases for each successive generation on Event 176. The best parameter values (BPV) were used to simulate the most likely evolutionary trajectory in northeast Spain, and a range of parameters were used to investigate possible alternative trajectories (Table 1) [25–38]. Additional details on these and all other parameter values are in the S1 File (Tables B-I and Figs E-G in S1 File).

**Table 1:** Parameter values used in resistance evolution model.

Parameter		BPV	Value Range	Source
**Environment**				
Proportion Event 176 *Bt* maize	*Q*_*1*_	Historical values (HV)	0–0.138	SI
Proportion late-season Event 176	*Q*_*1l*_	HVs	0.01	SI
Proportion Mon 810 *Bt* maize	*Q*_2_	HVs to 0.80	0–0.8	SI
Proportion late-season Mon 810	*Q*_2*l*_	HVs	0–0.75	SI
Proportion non-*Bt* maize	*Q*_3_	0.2 to HVs	0.2–0.948	SI
Proportion of maize rotated to another crop		0.45		SI
Proportion of *Bt* maize rotated to non-*Bt* maize		0.00		SI
Proportion of non-*Bt* maize rotated to *Bt* maize		0.00		SI
Refuge compliance		HVs	0.10–0.95	SI
**Population**				
Proportion of adults leaving field	*r*_*iag*_			
*i* = all, *a* = *v*, *g* = 1	*r*_*iv*1_	0.05	0.05–1.0	25, 26, 27
*i* = all, *a* = *f*, *g* = 1	*r*_*if*1_	1.0		25, 26, 27
*i* = all, *a* = *m*, *g* = 1	*r*_*im*1_	1.0		25, 26, 27
*i* = all, *a* = *v*, *g* = 2	*r*_*iv*2_	0.02	0.02–1.0	25, 28, 29, 30
*i* = all, *a* = *f*, *g* = 2	*r*_*if*2_	0.04	0.04–1.0	25, 28, 29, 30
*i* = all, *a* = *m*, *g* = 2	*r*_*im*2_	1.0		25, 28, 29, 30
*i* = all, *a* = *v*, *g* = 3	*r*_*iv*3_	0.02	0.02–1.0	SI
*i* = all, *a* = *f*, *g* = 3	*r*_*if*3_	1.0		SI
*i* = all, *a* = *m*, *g* = 3	*r*_*im*3_	1.0		SI
Relative preference for field *i*	*s*_*iag*_			
*i* = all, *a* = all, *g* = 1	*s*_*ia1*_	1/3		SI
*i* = all, *a* = all, *g* = 2	*s*_*ia2*_	1/3		SI
*i* = 1, *a* = all, *g* = 3	*s*_*1a3*_	*Q*_*1l*_/3*Q*_1_		SI
*i* = 2, *a* = all, *g* = 3	*s*_*2a3*_	*Q*_2*l*_/3*Q*_2_		SI
*i* = 3, *a* = all, *g* = 3	*s*_*3a3*_	(1- (*s*_*1a3*_+ *s*_*2a3*_)/3*Q*_3_		SI
Fecundity x DI survival	*F*_*ig*_			
*i* = all, *g* = 1	*F*_*i*1_	51.3		31, SI
*i* = all, *g* = 2	*F*_*i*2_	37.2		31, SI
*i* = all, *g* = 3	*F*_*i*3_	stochastic	0.0–18.3	31, SI
Diapause rate, *g* = 2		stochastic	0.10–0.80	32, SI
DD survival, *g* = 1	*a*_1_	0.1		SI
	*b*_1_	0.75		SI
DD survival, *g* = 2, 3	*a*_2_	0.11		33, SI
	*b*_2_	0.999		33, SI
Overwintering survival	*s*_*w*_	0.104		33, SI
**Genetic**				
Initial *R* allele frequency	*p*_0_	0.0015	0.0015–0.0029	24
Survival of *RR*, *i* = all, *g* = all	*L*_*ig*_	1.0		SI
Survival of *SS*	*k*_*ig*_			
*i* = 1, *g* = 1	*k*_11_	0.0001		14, 34–38, SI
*i* = 1, *g* = 2	*k*_12_	0.3		14, 34–38, SI
*i* = 1, *g* = 3	*k*_13_	0.7		14, 34–38, SI
*i* = 2, *g* = all	*k*_2*g*_	0.0001		SI
*i* = 3, *g* = all	*k*_3*g*_	1.0		SI
Heterozygosity	*h*_*ig*_			
*i* = 1, *g* = 1	*h*_11_	0.001		14, 34–38, SI
*i* = 1, *g* = 2	*h*_12_	0.3		14, 34–38, SI
*i* = 1, *g* = 3	*h*_13_	0.5		14, 34–38, SI
*i* = 2, *g* = all	*h*_2*g*_	0.001		SI
*i* = 3, *g* = all	*h*_3*g*_	0.5		SI
Assortative mating, *g* = all	*α*_*g*_	0.00	0.00–0.04	SI

BPV = best parameter values; SI = See S1 File (Supporting Information); DI survival = density-independent survival; DD survival = density-dependent survival; *i* = field type (1 = Event 176, 2 = Mon 810, 3 = non-*Bt*); *a* = adult type (*v* = virgin female, *f* = mated female, *m* = male), *g* = generation (1–3).

### Model Experiments

Although the model structure and parameterization were designed to provide the best predictions of the time to resistance, models are more useful for understanding how the relative time to resistance changes as parameter values change. Because of their heuristic nature, these kinds of investigations of model behaviour can be considered to be thought experiments, answering questions like “What if the parameters were different?” We varied the following parameter values comparing the simulation results to the BPV model: initial *R* allele frequency, female dispersal, Bt maize adoption rate, spatial structure of the refuge (and degree of assortative mating), external colonization and interactions among the parameters (see SI for additional details).

All simulations began with a “warm-up” period to remove artifacts from initial density transients. First, populations of *S*. *nonagrioides* were simulated on landscapes that had no *Bt* maize with the initial resistance allele frequency until the population densities reached equilibrium (no selection). This also generated an equilibrium distribution of the *R* allele (equal to the initial frequency in all patch types). After the populations reached equilibrium densities, *Bt* maize was introduced using the varieties and rates actually experienced in northeast Spain during the past 17 years. In addition, based on the actual weather data from northeast Spain, we estimated a diapause rate for the second generation and an end-of-the-season mortality rate for the third generation for the past 17 years (Table F in S1 File). Simulations continued until the average resistance allele frequency across all patch types exceeded 0.5, which was recorded as the number of years to resistance. Because success of the third generation was stochastic, we ran 1000 replications of each parameter combination and report the mean and standard deviations of these simulations.

## Results and Discussion

### Sustained efficacy of *Bt* maize for 16 years

**Fig 2:**
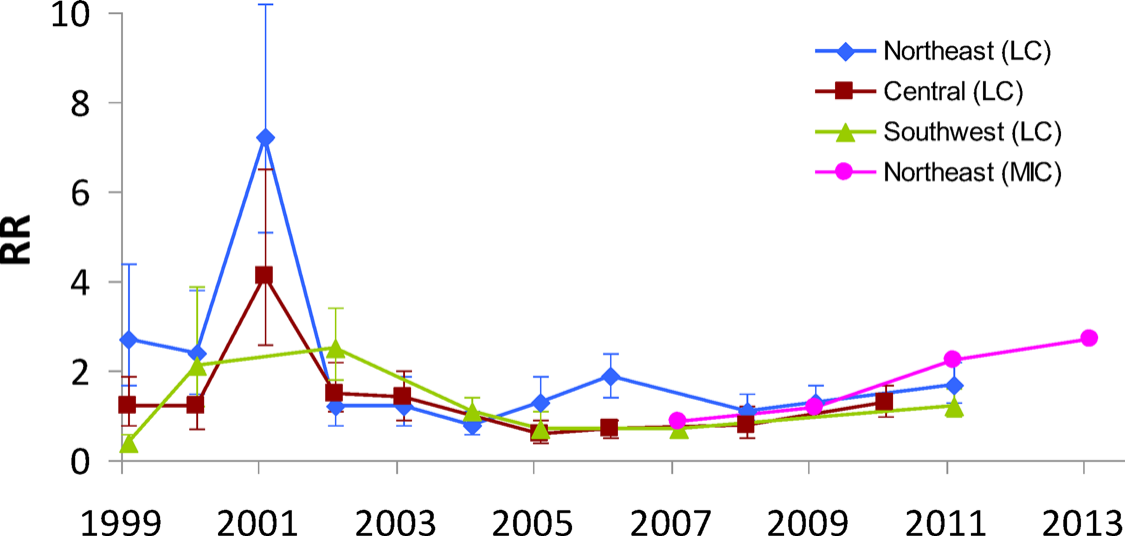
Susceptibility to Cry1Ab of Spanish field populations of *Sesamia nonagrioides*. Resistance Ratios (RR) with respect to a susceptible laboratory strain were calculated from: LC_50_ data of field populations from northeast, central and southwest Spain in the period 1999–2011 (see Table A in S1 File); and MIC_50_ data reported in the resistance monitoring programme undertaken by Monsanto Europe S.A. for field populations from northeast Spain in the period 2007–2013 (in EFSA, 2015 [18]). Error bars correspond to confidence limits at 95% for RR values calculated from LC_50_ values.

Available data revealed no shift in susceptibility to Cry1Ab in Spanish field populations of *S*. *nonagrioides* after 16 years (32–48 generations) of cultivation of *Bt* maize in this hotspot. A post-market resistance monitoring program was initiated in 1998, allowing us to establish a baseline and to measure the natural variations in susceptibility of field populations of *S*. *nonagrioides* [39]. Thereafter, susceptibility to Cry1Ab has been monitored every one or two years in the most representative Spanish Bt maize production areas. The results obtained with dose-probit analysis (see Table A in S1 File) demonstrate that the LC_50_s of field populations and the laboratory susceptible strain have remained within a narrow range during the period 1999–2011. Resistance ratios in northeast Spain showed no clear trends, being always below 4 except for an occurrence of 7 in 2001, and were similar to those found in regions with much lower adoption rates (central and southwest Spain) (Fig 2). A resistance monitoring program, using a different batch of Cry1Ab toxin was undertaken by Monsanto Europe S.A. for field populations from northeast Spain in the period 2007–2013 [18]. Interestingly, when comparing MIC_50_ values of the northeast population with respect to the laboratory susceptible population over time (Fig 2), no shifts in the susceptibility of field populations were reported. Moreover, an early alert system, based on farmer complaints due to unexpected plant damage, was included in this monitoring programme. No control failures against corn borers have been yet reported from any of the Spanish *Bt* maize areas.

### Factors Affecting the Rate of Resistance Evolution

**Fig 3:**
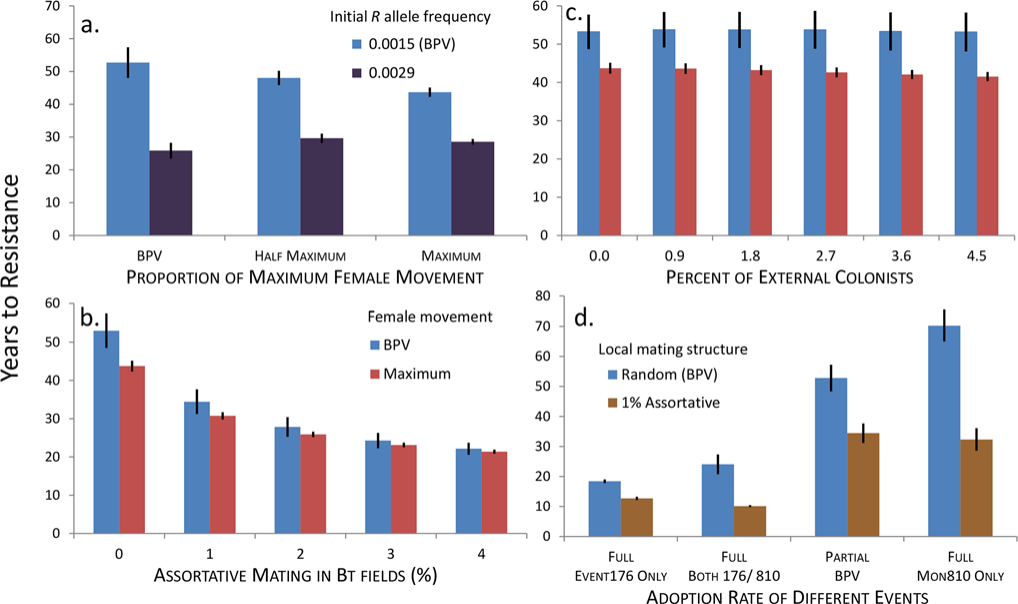
Predicted rate of resistance evolution for *Sesamia nonagrioides* in northeast Spain. Mean years to resistance (*R* allele frequency ≥ 0.5) for the interactions a) between amount of female movement and initial *R* allele frequency (BVP indicates the best parameter values, including the level of non-compliance to the refuge recommendation; Maximum: all females leave natal site before mating and leave mating site before ovipositing; Half Maximum: female movement parameters are halfway between BPV and Maximum); b) between local assortative mating in Bt fields and maximum female movement (initial *R* allele frequency = 0.0015); c) between level of external colonization and maximum female movement (legend as in 6b) (initial *R* allele frequency = 0.0015) d) between adoption rate of different Bt maize events and local assortative mating in Bt fields (Full: 80% adoption from year 1; Partial: observed adoption rates to 2014 and full adoption thereafter; Both 176/810: sum of adoption of Event 176 and MON 810 is 80% from year 1, and the proportion of each equals the observed proportion to 2014 and only MON 810 thereafter; initial *R* allele frequency = 0.0015); error bars are standard deviations of 1000 random simulations.

Overall, the factors that could delay resistance evolution (time to an *R* allele frequency that exceeds 0.5) dominated those that could accelerate it. We investigated the role of poor refuge compliance by using the actual estimated use of *Bt* maize and refuges in northeast Spain (Fig 1 and Table H in S1 File). For two different initial allele frequencies (northeast Spain: 0.0029; EU: 0.0015, see SI for additional details), we found that even with the observed poor refuge compliance, resistance evolution would take 26–53 years (Fig 3A, BPV female movement).

Female *S*. *nonagrioides* have limited pre-mating and post-mating dispersal [25]. Because limited female dispersal is predicted to greatly reduce the rate of resistance evolution [21], we modelled the effect of increased levels of female *S*. *nonagrioides* movement on resistance evolution up to the maximum movement possible. Increased movement reduced the time to resistance, but quantitatively, the effect was small, and even at the maximum possible movement, the projected time to resistance was still 43 years, only 10 years faster than BPV (Fig 3A). At the higher initial *R* allele frequency, the effect of female movement virtually disappeared (Fig 3A). Limited female movement reduced the rate of resistance evolution, but was not a primary factor influencing the durability of *Bt* maize in northeast Spain.

In addition to the amount of refuge, the location of refuges is considered critical to delay the evolution of resistance [20, 40]. By ensuring that refuges are located near *Bt* fields, there can be sufficient mixing of the population so that resistant moths are likely to mate with susceptible moths, thereby delaying resistance evolution. Resistant females emerging from a *Bt* maize field will mate with males that come from other fields, whereas resistant males will fly to adjacent fields to mate with sedentary susceptible females [25]. If refuges are too far away, resistant females will tend to mate with resistant males, with local positive assortative mating. To evaluate the role of the spatial structure of refuge, we allowed small amounts of assortative mating only in *Bt* maize fields (see SI for justification). With even 1% positive assortative mating, the projected time to resistance drops by about 30% to 34 years (Fig 3B). With 4% assortative mating, this drops to only 22 years. Thus properly located refuges, whether by happenstance or plan, have been critical for maintaining the durability of *Bt* maize for *S*. *nonagrioides*.

In Australian cotton, *Helicoverpa armigera* evolved resistance to all major classes of insecticides [41], while it never occurred in the congener, *H*. *punctigera*. The main reason accounting for this difference was that there was a large influx of unselected *H*. *punctigera* each year from another geographic region followed by mating between colonists and residents, while this did not occur for *H*. *armigera* [42, 43]. It is unlikely that this was happening in northeast Spain, because *S*. *nonagrioides* is a sedentary species [17], so long-distance migration is probably uncommon. However, even if it were to occur, colonists would likely originate from the south, either southern Spain or northwestern Africa, and ride the prevailing winds to northeast Spain. Consequently, colonists would probably arrive earlier than emergence of the overwintering population in northeast Spain, because emergence at the source locations would happen much earlier. So there might be little mating between colonists and overwintered residents, and colonists would contribute only their offspring to the first generation with genetic mixing occurring in the second and third generations. In this case, the model showed that even a large influx of colonists would have no discernible effect on the rate of resistance evolution (Fig 3C).

The initial low adoption of *Bt* maize in northeast Spain (Fig 1) reduced selection for resistance, increasing the time to resistance. To evaluate the effect of low adoption, we modelled the evolutionary process with full adoption (80% *Bt* maize in northeast Spain) from the first years of commercial use under the assumption that Event 176 and MON 810 split the market at their observed shares of the *Bt* maize market. Event 176 would allow heterozygote survival during the 2^nd^ and 3^rd^ generations (Table E in S1 File), and the switch to MON 810 would maintain high-dose selection on all three generations (see SI for additional details). We also considered the possibilities that only Event 176 or only MON 810 was available and fully adopted. Low initial adoption, which allowed the timely transition from Event 176 to MON 810, was critical for the lack of detectable resistance in this EU hotspot (Fig 3D). The BPV projection indicates that resistance would evolve in 53 years. If there had been full adoption during the first years of commercialization, resistance would have occurred in only 24 years (Fig 3D), so low adoption provided 29 more years before resistance would be expected to occur under BPV conditions. If in addition, Event 176 had not been replaced by MON 810, resistance would be predicted to be widespread in 18 years (2016). Instead of accounting for the “paradox” of no observed resistance, we would be accounting for sporadic resistance failures! If only MON 810 had been available and fully adopted, resistance was projected in 70 years, significantly longer than the 53 years projected under the present conditions. Clearly, the EU policy to take Event 176 off the market was an important factor that delayed the evolution of resistance.

## Conclusions

Our results suggest that if two conditions hold, 1) the initial *R* allele frequency was not higher than 0.0015 and mating has been and continues to be locally random, then Cry1Ab *Bt* maize is likely to last an additional 20+ years in northeast Spain. Although some may consider this a long time, it is important to confirm these key assumptions and to implement strategies that postpone resistance further into the future. For example, if refuges had been located so that only 2% assortative mating occurred in *Bt* fields, the expected time to resistance would be reduced to only 10 years from now (Fig 3B). Clearly, obtaining more accurate estimates of *R* allele frequency by improving rearing methods to enable routine use of F_2_ screens [44], and determining that mating is locally random are high priorities to improve these projections of resistance evolution.

Pyramiding multiple toxins may allow a reduction in the size of the refuge [45, 46] and/or postpone resistance further into the future if there is no cross-resistance between the toxins [47] and the refuge is large enough to sustain a viable population [48]. A number of single and stacked maize events expressing different toxins targeting corn borers are available in the USA, but only those expressing Cry1F are currently under consideration for cultivation in EU (http://www.gmo-compass.org/eng/gmo/db/). Maize hybrids expressing Cry1F toxin provide good control of European populations of *S*. *nonagrioides* [49] and *Ostrinia nubilalis* [50], another major maize pest in Spain. However, cross-resistance between Cry1Ab and Cry1F toxins cannot be excluded [51]. Competition experiments indicated that, in *S*. *nonagrioides*, Cry1F and Cry1Ab have different high-affinity binding sites, though Cry1Fa binds to Cry1Ab binding site with very low affinity and vice versa [52]. In addition, though low levels of cross-resistance between Cry1Ab and Cry1F have been reported for *O*. *nubilalis* [53], partial cross-recognition of the binding sites for these two toxins was found in this species [54]. Thus, substitution of single events with stacks should be carefully managed to fully profit from them [55]. Pyramided maize may also contribute to better control of other secondary lepidopteran pests, such as *Mythimna unipuncta*, whose potential to develop resistance to Cry1Ab-expressing maize cultivars has been recently demonstrated [56, 57]. Although reducing the refuge with pyramids might improve refuge compliance, current compliance in northeast Spain is over 90% (Table H in S1 File), and research in the US suggested that a 20% refuge was acceptable to farmers [58], so reducing the size of the refuge would probably result in only marginal improvements in compliance in Spain and would reduce the durability of the pyramid. Thus pyramiding Cry1Ab and Cry1F while maintaining the present 20% refuge requirement might provide better control of some primary and secondary pests and help to achieve long-term durability of Bt maize.

## Supporting Information

S1 File Includes 9 Tables and 7 Figures. Study System in Spain, Formulation and Parameterization of Model, and Simulation Experiments.
(PDF)

S2 File Geographic coordinates for the sampling sites. (PDF)
