# Correction: Sixteen Years of *Bt* Maize in the EU Hotspot: Why Has Resistance Not Evolved?

**DOI:** 10.1371/journal.pone.0160294

**Published:** 2016-07-25

**Authors:** Pedro Castañera, Gema P. Farinós, Félix Ortego, David A. Andow

S2 File appears incorrectly in the published article. Please see the correct S2 file and its caption here.

## Supporting Information

S2 File Geographic coordinates for the sampling sites. (PDF)
