# Supplementary material for: Sixteen Years of Bt Maize in the EU Hotspot: Why Has Resistance Not Evolved?
Source: PLoS One. 2016 May 4;11(5):e0154200. doi: 10.1371/journal.pone.0154200 (PMC4856266; doi:10.1371/journal.pone.0154200)
Supplement: S1 File — Study System in Spain, Formulation and Parameterization of Model, and Simulation Experiments. (PDF) [file pone.0154200.s001.pdf]

S1 File (Supporting Information)

Sixteen years of Bt maize in the EU hotspot without resistance: Why has resistance not evolved?

Study System in Spain, Formulation and Parameterization of Model, and Simulation Experiments

Pedro Castañera, Gema P. Farinós, Félix Ortego, David A. Andow

1. Description of Maize Study System in Spain

The most damaging pest of maize in Spain is the Mediterranean corn borer (MCB) *Sesamia nonagrioides* Lefèbvre (Lepidoptera: Noctuidae). It feeds on host plants mainly belonging to Gramineae, including weeds (*Sorghum halepense* and *Setaria* spp) and cultivated plants (*Sorghum bicolor* and *Oryza sativa)*. In the maize-growing region of northeast Spain it is functionally monophagous on maize because weeds are well-managed in maize in the region and other crop host plants are rare.

It completes a variable number of generations per year depending on latitude, ranging from two generations in northwest Spain [1] to two generations and a partial third on maize in southern France and northeast Spain (Fig A) [2-5], and up to four in Morocco and Portugal [6-7]. The size of the 3rd generation depends on the proportion of 2nd generation larvae that enter diapause. In some years, 3rd generation larvae probably have enough time to enter diapause as last instars. Diapause is induced by short day length during the 1st and 2nd instars during early August [5] and the induction rate is modified by temperature and maize development stage [8,9]. Diapausing larvae feed and can undergo an indeterminate number of supernumerary larval molts during the winter [10, 11]. Non-diapausing larvae typically have six instars. Overwintered larvae emerge as adults during late April to late June [12].

Females of the first flight oviposit between the stem and leaf sheath on the leaves of maize, and neonates begin boring into the stem shortly after hatching. The second flight occurs between mid-July and late August, and a third flight occurs from mid-September to mid-October [12]. Females of the second flight probably oviposit between the stem and leaf sheath in the middle part of the plant, and neonates begin boring into the stem shortly after hatching. Females in the third flight will often oviposit on *Bt* and non-*Bt* late sown maize (June-July) as second crop, long-season maize, and some

forage non-*Bt* maize. In some years, there is enough heat at the end of the year that allows the third generation to complete development on forage maize. The first generation is particularly devastating, because larvae tunnel throughout the young maize stem and can kill the plant. Yield loss can be substantial [3, 13].

Fig A. Phenology of *Sesamia nonagrioides* and maize growth and development in northeast Spain. For *S. nonagrioides* flights, the thick bar is the range of variation for the “peak” or 50% point of the flight, and the thin lines are for 10%-90% of the flight. Time when day length conditions are appropriate for diapause induction (*) and breaking of diapause (**). Based on 10 years data [12].

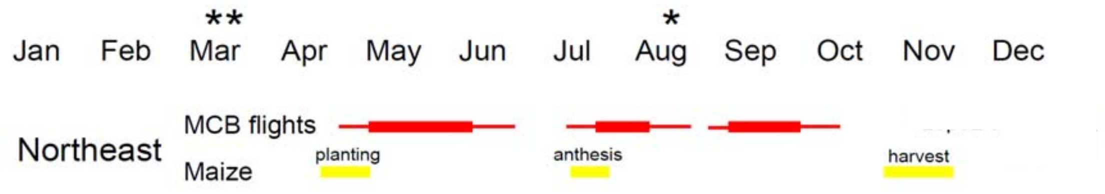

Maize cultivation in northeast Spain is mostly located on the Ebro valley (Cataluña: Lleida; and Aragón: Zaragoza and Huesca; with continental climate), although there is some maize cultivation close to the Mediterranean Sea (Cataluña: Girona, Mediterranean climate) (Fig B). The average size of maize farms (maize surface/number of farms) was 7.2 ha for Cataluña and 6.3 for Aragón in 2005 [14, 15] and 6.9 ha for Cataluña and 9.0 for Aragón in 2009 [16]. However, each maize farm is usually formed by several maize fields.

Fig B. Cultivated surface (ha) of maize in municipalities in northeast Spain [16].

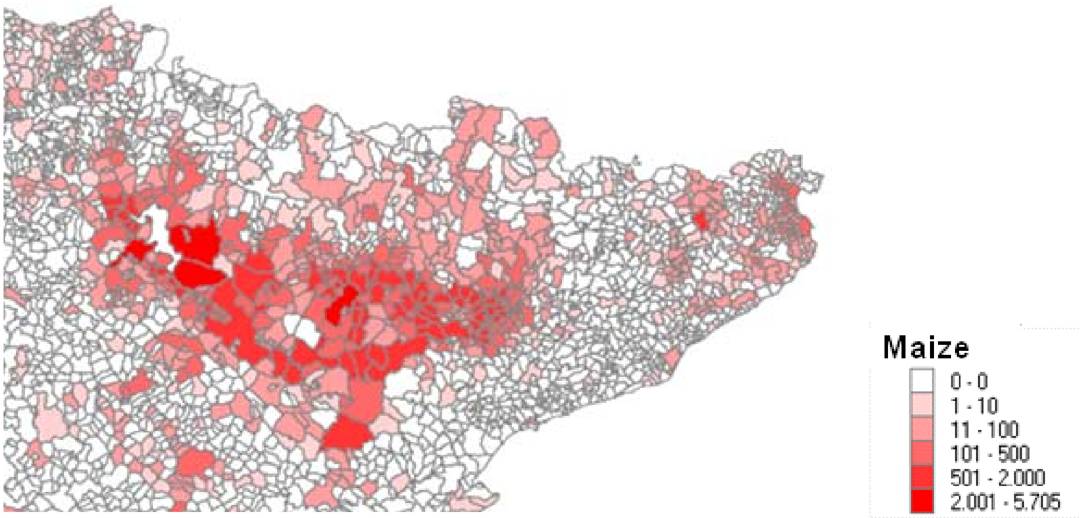

Larger fields are associated with center pivot irrigation in this region (Fig C), but most fields are much smaller. The maize landscape was characterized (growing season 2004) in two areas in Cataluña: Térmens (Lleida) and Pla de Foixà (Girona) [17]. The total area studied in Térmens was 300 ha, whereas in Pla de Foixà the area was 400 ha. In both regions, *Bt* maize (Event 176 and MON 810) and conventional maize fields coexisted with other crops (cereals, fruit trees, etc.), and were characterized by the small size of the fields (0.5–5 ha, with a mean of about 2 ha). In many cases, maize fields were in close proximity to each other (1-10 m)

Fig C. Fields around Candasnos, Huesca, Spain. The large circles are 500m in diameter.

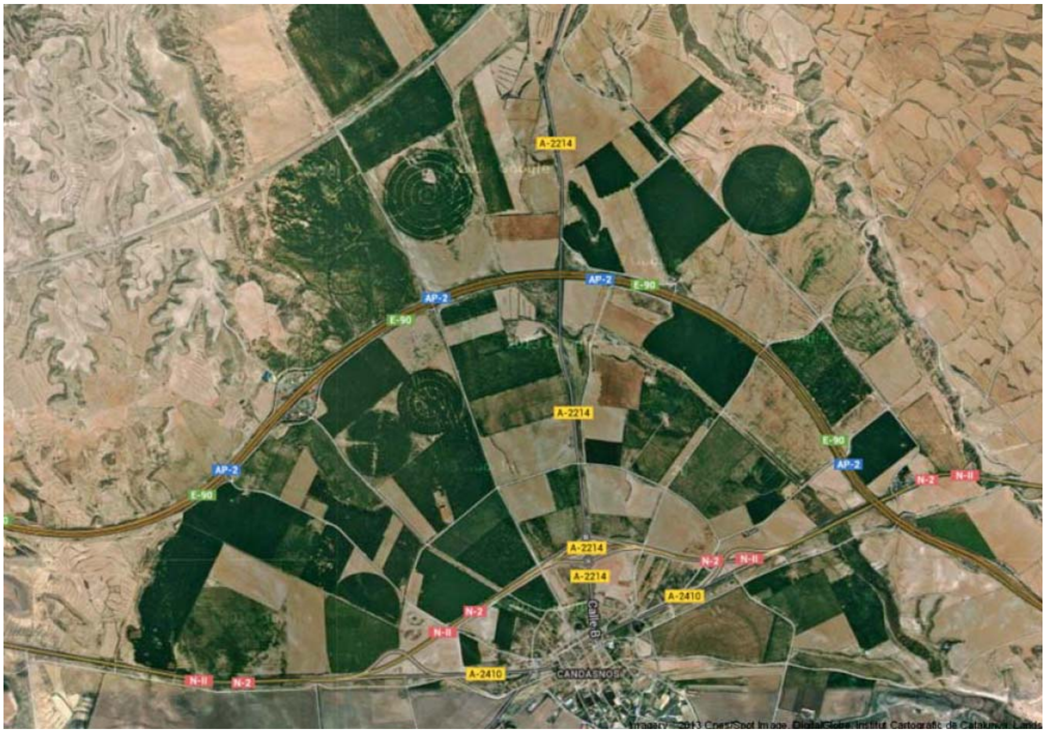

The susceptibility to Cry1Ab toxin of field populations of *S. nonagrioides* from Ebro Valley and from two other representative’s Spanish maize growing areas was monitored between 1999 and 2011 as part of the post-market monitoring program established by the “Ministerio de Agricultura, Alimentación y Medio Ambiente” (http://www.magrama.gob.es/es/),. The probit analysis of those populations and of a laboratory strain is shown in Table A.

Table A. Susceptibility of *Sesamia nonagrioides* neonates to the insecticidal toxin Cry1Ab, based on lethal concentrations (LC).

Maize growing area for larvae collection[a]	Year	Type of maize [b]	n [c]	Slope ± SE	χ^2	d.f.	LC_{50} [d] (CL 95%)	LC_{90} [d] (CL 95%)
Northeast Spain	1999 †	T	429	2.4 ± 0.4	8.8	7	23 (14-31)	78 (55-150)
	2000 †	T	336	3.1 ± 0.5	1.4	5	20 (15-25)	52 (41-77)
	2001 †	T	432	2.0 ± 0.3	3.9	7	34 (27-42)	156 (111-282)
	2002 †	T	384	1.6 ± 0.2	12.5	10	11 (7-16)	76 (46-184)
	2003 ‡	T	639	1.8 ± 0.2	46.8	33	22 (15-29)	114 (84-178)
	2003 ‡	NT	224	1.7 ± 0.3	21.7	12	16 (7-27)	88 (51-232)
	2004 ‡	R + NT	576	2.2 ± 0.2	20.5	16	22 (17-26)	81 (64-112)
	2005 ‡	T	670	2.8 ± 0.2	33.8	19	30 (24-37)	88 (70-124)
	2006	R	669	2.4 ± 0.2	33.6	19	29 (22-35)	98 (74-148)
	2008	R	672	1.6 ± 0.2	31.4	19	30 (21-40)	183 (119-368)
	2009	R	676	1.8 ± 0.2	33.7	19	23 (18-30)	122 (85-207)
	2011	R	671	2.5 ± 0.2	39.8	19	40 (33-47)	131 (102-191
Central Spain	1999	T	672	1.8 ± 0.2	20.1	19	10 (8-13)	53 (41-75)
	2000	T	579	2.0 ± 0.3	332.6	22	10 (6-14)	45 (33-76)
	2000	NT	432	1.7 ± 0.4	10.2	7	36 (14-66)	195 [e]
	2001 †	T	432	1.9 ± 0.3	2.8	7	19 (13-26)	94 (69-148)
	2001	NT	336	2.8 ± 0.4	2.8	5	18 (15-21)	51 (41-71)
	2002 †	T	384	1.5 ± 0.2	31.5	14	15 (9-23)	106 (57-356)
	2003 ‡	T	592	2.2 ± 0.3	40.6	28	25 (18-31)	95 (68-175)
	2003 ‡	NT	671	1.8 ± 0.2	19.3	19	23 (18-28)	117 (86-180)
	2005 ‡	T	895	2.4 ± 0.2	43.6	25	14 (11-16)	46 (37-60)
	2006	R	670	2.0 ± 0.2	25.9	19	10 (8-13)	44 (34-60)
	2008	R	644	1.2 ± 0.4	45.1	19	21 (11-35)	255 (124-1048)
	2010	R	672	2.0 ± 0.2	61.7	33	30 (23-39)	131 (94-220)
Southwest Spain	1999	T	144	2.6 ± 0.6	10	7	3 (2-5)	10 (6-51)
	2000 †	T	333	3.1 ± 0.7	10.7	5	18 (6-28)	45 [e]
	2000	NT	384	1.4 ± 0.3	3.4	6	19 (11-28)	151 (88-483)
	2002	T	622	2.5 ± 0.3	28.2	11	24 (16-32)	81 (57-164)
	2003 ‡	NT	672	2.0 ± 0.1	22.2	19	21 (18-26)	94 (75-126)
	2004 ‡	T	672	1.7 ± 0.1	27.4	19	28 (21-36)	165 (119-256)
	2004 ‡	NT	672	2.3 ± 0.2	22.4	19	24 (18-31)	89 (70-121)
	2005 ‡	T	893	2.5 ± 0.2	71.9	26	17 (12-21)	54 (41-79)
	2005 ‡	NT	767	2.3 ± 0.2	139.3	22	12 (6-19)	45 (29-102)
	2007	R	672	2.0 ± 0.2	39.7	19	18 (14-23)	75 (55-114)
	2011	R	890	2.0 ± 0.2	73.5	26	29 (22-38)	131 (91-241)
Laboratory	2000 †	-	304	2.8 ± 0.5	9.5	8	8 (5-11)	24 (18-41)
	2001 †	-	336	3.0 ± 0.4	9.9	12	5 (4-6)	13 (10-17)
	2002 †	-	384	3.2 ± 0.4	13.0	12	10 (7-12)	25 (20-36)
	2003 ‡	-	336	1.9 ± 0.2	17.1	19	18 (13-24)	86 (63-129)
	2004 ‡	-	573	2.5 ± 0.3	33.2	16	26 (18-34)	87 (65-147)
	2005 ‡	-	336	1.8 ± 0.2	17.5	19	23 (16-30)	119 (86-185)
	2006	-	672	2.1 ± 0.2	30.6	19	15 (12-20)	61 (47-87)
	2008	-	672	2.7 ± 0.3	16.2	19	26 (22-30)	79 (65-101)
	2009	-	672	2.0 ± 0.2	54.9	19	18 (12-24)	77 (53-145)
	2010	-	668	2.1 ± 0.2	28.4	19	23 (18-29)	97 (72-147)

[a] The three main Spanish maize growing regions considered were: northeast (Cataluña and Aragon), central (Madrid and Castilla-La Mancha) and southwest (Extremadura and Andalucía).

[b] Type of maize where last instar larvae were collected: *T* is transgenic maize, var. Compa CB (event Bt176, Syngenta), *NT* is non-transgenic maize and *R* is a refuge of non-transgenic maize adjacent to MON 810 maize.

[c] Number of neonates tested in the bioassays.

[d] Concentrations are expressed in ng Cry1Ab/cm^2.

[e] 95% CL could not be estimated because the coefficient g was >0.5 at the 95% probability level.

† Data from [18].

‡ Data from [19].

There are also data from field populations in northeast Spain during the period 2007-2013 from a resistance monitoring program undertaken by Monsanto Europe S.A. (in EFSA 2015 [20]). In this case, moulting inhibition concentrations (MIC_{50}) were calculated using a batch of Cry1Ab toxin that was different from that used in our LC_{50} data. The results obtained were 14 (2007), 22 (2009), 20 (2011) and 19 ng Cry1Ab/cm^2 (2013) for the field population from northeast Spain, and 16 (2007), 19 (2009), 9 (2011) and 7 ng Cry1Ab/cm^2 (2013) for a laboratory strain [20]

2. Formulation of Simulation Model

This model follows closely previous models [21-24] but we provide a complete derivation here for the readers' convenience (Fig D). The model is a patch model with three patch types, but generalizes for any number of patch types. We designate patch 1 for Event 176 *Bt* maize, patch 2 for MON 810 *Bt* maize and patch 3 for non-*Bt* refuge maize and other refuge plants. Nearly all of the refuge plants in northeast Spain are non-*Bt* maize.

Fig D. Diagram of model structure for a) an annual cycle and b) within one of the three generations.

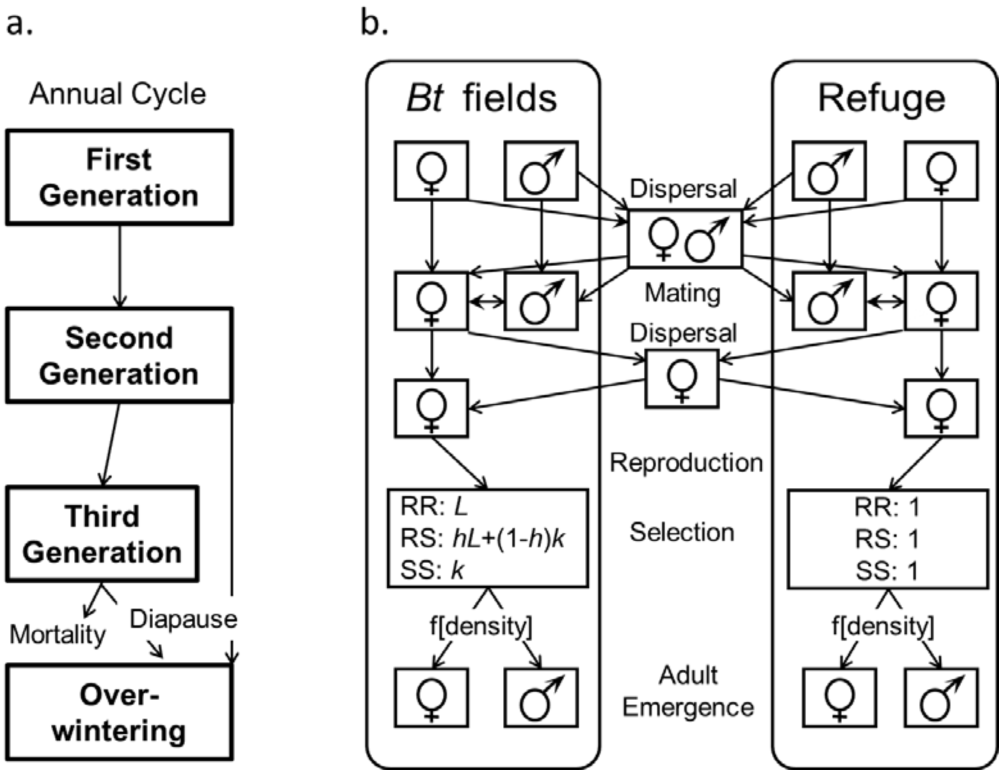

Let $\mathbf{P}(t) = \begin{pmatrix} p_1(t) \\ p_2(t) \\ p_3(t) \end{pmatrix}$ be the vector of frequencies of the resistance allele in Event 176 *Bt*, Mon 810 *Bt* and refuge fields in generation *t* ($p_1(t)$, $p_2(t)$ and $p_3(t)$, respectively). Similarly, let $\mathbf{X}(t) = \begin{pmatrix} x_1(t) \\ x_2(t) \\ x_3(t) \end{pmatrix}$ be the vector of the number of *S. nonagrioides* adult females in the three field types in generation *t* after emergence and before dispersal. In these simulations, we assume that the sex ratio at emergence is 50%, so **X**(*t*) is also the number of *S. nonagrioides* males before dispersal.

The movement of males, virgin and mated females among *Bt* and refuge fields is given by the matrices $\mathbf{M_a}$ where $\mathbf{a} \in \{v, f, m\}$, *v* are virgin females, *f* are mated females and *m* are males, whose ij^{th} elements (*i*, *j* = 1 for Event 176 *Bt* fields, 2 for MON 810 *Bt* fields and 3 for refuge fields) give the proportion of the male and female populations in fields of type *i* that moved from fields of type *j*. Specifically,

$$\mathbf{M_a} = \left(\frac{z_{ij}}{Norm_i} \right) = \begin{pmatrix} \frac{z_{11}}{z_{11}+z_{12}+z_{13}} & \frac{z_{12}}{z_{11}+z_{12}+z_{13}} & \frac{z_{13}}{z_{11}+z_{12}+z_{13}} \\ \frac{z_{21}}{z_{21}+z_{22}+z_{23}} & \frac{z_{22}}{z_{21}+z_{22}+z_{23}} & \frac{z_{23}}{z_{21}+z_{22}+z_{23}} \\ \frac{z_{31}}{z_{31}+z_{32}+z_{33}} & \frac{z_{32}}{z_{31}+z_{32}+z_{33}} & \frac{z_{33}}{z_{31}+z_{32}+z_{33}} \end{pmatrix} \quad \text{(M1)}$$

where z_{ij} are the numbers of adults starting in field type *j* and ending up in field type *i*, and includes both those that stay in their natal field and those that move to another field (including fields similar to their natal field). The z_{ij} are normalized by the total number ending in field type *i*, so the rows sum to 1. The numbers of adults starting in field type *j* and ending in field type *i* are

$$\mathbf{Z_{ag}} = (z_{ij})_{\mathbf{ag}} = \mathrm{Diag}[(1-\mathbf{r_{ag}}) \times \mathbf{X}(t)] + \left(\frac{\mathbf{r_{ag}} \times \mathbf{s_{ag}} \times \mathbf{Q}}{\mathbf{s_{ag}} \cdot \mathbf{Q}} \otimes \mathbf{X}(t) \right) \quad \text{(M2)}$$

where $\mathbf{r_{ag}} = (r_{ag1}, r_{ag2}, r_{ag3})$ are the proportions of adult type **a** in generation **g** that disperse from the three field types, with $\mathbf{g} \in \{1,2,3\}$ because there are two and sometimes a third generations of *S. nonagrioides* per year in Spain; $\mathbf{s_{ag}} = (s_{ag1}, s_{ag2}, s_{ag3})$ are the movement preferences of adults for the three field types, which is measured as the proportion choosing to move to each field type when the three field types are equally abundant; and $\mathbf{Q} = (Q_1, Q_2, Q_3)$ is the proportion of area planted to the three field types and may vary with time. The mathematical operators are as follows: $\mathbf{A} \cdot \mathbf{B}$ is the dot product (aka scalar or inner product) of two vectors or matrices of equal dimension; $\begin{pmatrix} a \\ b \end{pmatrix} \times \begin{pmatrix} c \\ d \end{pmatrix} = \begin{pmatrix} ac \\ bd \end{pmatrix}$ is the direct product (aka Hadamard or Schur product) of two vectors or matrices of equal dimension; and $\mathbf{A} \otimes \mathbf{B}$ is the Kronecker product of two vectors which produces a matrix, $\begin{pmatrix} a \\ b \end{pmatrix} \otimes \begin{pmatrix} c \\ d \end{pmatrix} = \begin{pmatrix} ac & ad \\ bc & bd \end{pmatrix}$. Diag is a function that takes a vector of length k and makes it into a diagonal matrix with dimensions k x k. The Diag function is used here to avoid lengthier matrix formulas. When $s_{agi} = 1/3$ (no preferential movement towards any fields), or when $Q_i = 1/3$, these expressions simplify substantially.

2.1. Dispersal and local non-random mating

Let α be the level of local assortative mating (within a field), such that $(1-\alpha)2pq$ is the frequency of heterozygotes in the offspring. This makes positive (negative) α be the proportional reduction (supplementation) of heterozygotes due to non-random mating. The local frequency of the genotypes in the next generation is

Genotype	SS	SR	RR
Frequency	$p\,(p + \alpha q)$	$2pq\,(1 - \alpha)$	$q\,(\alpha p + q)$

When $\alpha = 0$, the offspring frequencies correspond to local random mating as predicted by Hardy-Weinberg equilibrium. When $\alpha = 1$, there are no heterozygotes, and this is the greatest positive assortative mating possible. When $\alpha < 0$, mating is disassortative, and there is an excess of heterozygotes. Temporarily dropping the generations subscript, let $\mathbf{P}_{RR}$, $\mathbf{P}_{RS}$ and $\mathbf{P}_{SS}$ denote the frequency of *RR*, *RS*, and *SS* larvae laid the in the three field types under non-random mating:

$$
\begin{aligned}
\mathbf{P}_{RR} &= \mathbf{M}_{\mathbf{f}}\left(\left(\mathbf{M}_{\mathrm{v}}\mathbf{P}(t)\right)\times\left(\mathbf{M}_{\mathbf{m}}\mathbf{P}(t)\right)\right)+\\
&\quad(\alpha/2)\mathbf{M}_{\mathbf{f}}\left(\left(\left(1-\mathbf{M}_{\mathrm{v}}\mathbf{P}(t)\right)\right)\times\left(\mathbf{M}_{\mathbf{m}}\mathbf{P}(t)\right)+\mathbf{M}_{\mathrm{v}}\mathbf{P}(t)\times\left(1-\mathbf{M}_{\mathbf{m}}\mathbf{P}(t)\right)\right)\\
\mathbf{P}_{RS} &= (1-\alpha)\mathbf{M}_{\mathbf{f}}\left(\left(\left(1-\mathbf{M}_{\mathrm{v}}\mathbf{P}(t)\right)\right)\times\left(\mathbf{M}_{\mathbf{m}}\mathbf{P}(t)\right)+\mathbf{M}_{\mathrm{v}}\mathbf{P}(t)\times\left(1-\mathbf{M}_{\mathbf{m}}\mathbf{P}(t)\right)\right) \qquad \text{(M3)}\\
\mathbf{P}_{SS} &= \mathbf{M}_{\mathbf{f}}\left(\mathbf{M}_{\mathrm{v}}\left(1-\mathbf{P}(t)\right)\times\left(1-\mathbf{M}_{\mathbf{m}}\mathbf{P}(t)\right)\right)+\\
&\quad(\alpha/2)\mathbf{M}_{\mathbf{f}}\left(\left(\left(1-\mathbf{M}_{\mathrm{v}}\mathbf{P}(t)\right)\right)\times\left(\mathbf{M}_{\mathbf{m}}\mathbf{P}(t)\right)+\mathbf{M}_{\mathrm{v}}\mathbf{P}(t)\times\left(1-\mathbf{M}_{\mathbf{m}}\mathbf{P}(t)\right)\right)
\end{aligned}
$$

where, as above, × denotes the direct product of two vectors.

Equation (M3) can be explained as follows. Consider the top line, which has two parts. In the first part, $\mathbf{M_m P}(t)$ is the vector of resistance frequencies in males in all field types following male dispersal, while $\mathbf{M_v P}(t)$ gives the resistance allele frequencies of virgin females at the time of mating after they have dispersed. Thus, $\mathbf{M_v P}(t) \times \mathbf{M_m P}(t)$ is the frequency of homozygous *RR* eggs in all field types that would be laid by females prior to their post-mating movement**.** Before laying these eggs, however, females disperse among field types according to $\mathbf{M_f}$, so the distribution of homozygous *RR* eggs is $\mathbf{M_f}(\mathbf{M_v P}(t)\times\mathbf{M_m P}(t))$ under random mating. The second part is due to non-random mating. If $\alpha = 0$, the second part is 0 and mating is locally random. If $\alpha > 0$, mating is assortative. If $\alpha < 0$, mating is disassortative. Half of the heterozygote deficiency in line 2 contributes an *RR* genotype and half contributes an *SS* genotype. This non-random contribution to *RR* genotypes is $(\alpha/2)$ times the heterozygote frequency under random mating. Because $\mathbf{M_v P}(t)$ is the resistant allele frequencies in virgin females in all field types, 1- $\mathbf{M_v P}(t)$ is the susceptible allele frequency of virgin females in all field types. Thus, under random mating, $(1-\mathbf{M_v P}(t))\times\mathbf{M_m P}(t)$ would be the frequency of *RS* eggs contributed by an *S* allele from a female and an *R* allele contributed by a male. Similarly, $\mathbf{M_v P}(t)\times(1-\mathbf{M_m P}(t))$ would be the frequency of *RS* eggs contributed by an *R* allele from a female and an *S* allele from a male. Under non-random mating, the two *R* alleles do not contribute to heterozygotes, but instead become an extra *RR* homozygote. Before these eggs are laid, mated females must move according to $\mathbf{M_f}$, so the contribution to *RR* genotypes under non-random mating is $(\alpha/2)\ \mathbf{M_f}(((1-\mathbf{M_v P}(t))\times\mathbf{M_m P}(t))+(\mathbf{M_v P}(t)\times(1-\mathbf{M_m P}(t))))$. The remaining two lines are derived similarly for heterozygote and homozygous susceptible genotypes.

2.2. Selection

Selection for resistance is determined by the fitnesses of *RR*, *RS*, and *SS* larvae in the three field types. We track only alleles that convey resistance to MON 810 *Bt* maize. Event 176 *Bt* maize

expresses the same Cry toxin but at lower concentrations after anthesis, so any *R* allele giving resistance to MON 810 is likely to give resistance to Event 176.

GENOTYPE	SS	RS	RR
ABSOLUTE FITNESS	$f_{i,g,SS}$	$f_{i,g,RS} = h_{i,g}\, f_{i,g,RR} + (1\text{-}h_{i,g})\, f_{i,g,SS}$	$f_{i,g,RR}$
RELATIVE SURVIVAL	$k_{i,g}$	$L_{i,g}h_{i,g}+k_{i,g}(1\text{-}h_{i,g})$	$L_{i,g}$

where $L_{i,g}$ and $k_{i,g}$ are the respective survivals of resistant and susceptible homozygous genotypes in field *i* and generation *g*, and $h_{i,g}$ is the heterozygosity of resistance ($h_{i,g} = 0$ is recessive) in the three field types. In the *Bt* fields, $h_{i,g}$ is the heterozygosity of resistance, and in the refuge field, it is the heterozygosity of the cost of resistance. Letting $\mathbf{\Theta_{RR,g}}$, $\mathbf{\Theta_{RS,g}}$, and $\mathbf{\Theta_{SS,g}}$ denote the matrices whose i^{th} diagonal element gives the survivals of *RR*, *RS*, and *SS* larvae in fields of type *i*, during generation *g*,

$$\mathbf{\Theta}_{\mathbf{RR},g} = \begin{pmatrix} L_{1,g} & 0 & 0 \\ 0 & L_{2,g} & 0 \\ 0 & 0 & L_{3,g} \end{pmatrix}$$

$$\mathbf{\Theta}_{\mathbf{RS},g} = \begin{pmatrix} L_{1,g}h_{1,g} + k_{1,g}(1-h_{1,g}) & 0 & 0 \\ 0 & L_{2,g}h_{2,g} + k_{2,g}(1-h_{2,g}) & 0 \\ 0 & 0 & L_{3,g}h_{3,g} + k_{3,g}(1-h_{3,g}) \end{pmatrix} \quad \text{(M4)}$$

$$\mathbf{\Theta}_{\mathbf{SS},g} = \begin{pmatrix} k_{1,g} & 0 & 0 \\ 0 & k_{2,g} & 0 \\ 0 & 0 & k_{3,g} \end{pmatrix}$$

Let $\mathbf{W}_{RR}$, $\mathbf{W}_{RS}$, and $\mathbf{W}_{SS}$ denote the vectors of relative frequencies of *RR*, *RS*, and *SS* larvae surviving in *Bt* and refuge fields, so (dropping the generation subscript temporarily)

$$\mathbf{W}_{RR} = \mathbf{\Theta}_{\mathbf{RR}}\mathbf{P}_{RR}$$

$$\mathbf{W}_{RS} = \mathbf{\Theta}_{\mathbf{RS}}\mathbf{P}_{RS} \quad \text{(M5)}$$

$$\mathbf{W}_{SS} = \mathbf{\Theta}_{\mathbf{SS}}\mathbf{P}_{SS}$$

The change in the frequency of resistance alleles is given by the recursion equation

$$\mathbf{P}(t+1) = \left(\mathbf{W}_{RR} + \mathbf{W}_{RS}/2\right) \times \left(\mathbf{W}_{RR} + \mathbf{W}_{RS} + \mathbf{W}_{SS}\right)^{inv}, \tag{M6}$$

which normalizes the frequencies after selection, where *inv* denotes the inverse of the direct product (Hadamard or Schur product): $\begin{pmatrix} a \\ b \end{pmatrix}^{inv} = \begin{pmatrix} 1/a \\ 1/b \end{pmatrix}$.

The rate of resistance evolution (M6) depends generally on the population sizes in *Bt* and refuge fields, because population sizes appear in the matrices giving the movement of males and females among field types, $\mathbf{M_{mg}}$, $\mathbf{M_{vg}}$ and $\mathbf{M_{fg}}$.

2. 3. Population dynamics

The vector of numbers of male and female larvae within *Bt* and refuge fields following female dispersal and selection, $\mathbf{X}'(t)$, is given by

$$\mathbf{X}'(t) = \left(\mathbf{F_g D_{fg} D_{vg}}(\mathbf{X}(t))\right) \times \left(\mathbf{W}_{RR,g} + \mathbf{W}_{RS,g} + \mathbf{W}_{SS,g}\right) \tag{M7}$$

where $\mathbf{D_{vg}}$ is the matrix giving the rates of virgin female dispersal among field types, $\mathbf{D_{fg}}$ is the matrix giving the rates of mated female dispersal among field types, and $\mathbf{F_g}$ is fecundity times density independent (DI) survival in each field type for generation *g*.

$$\mathbf{D_{ag}} = \left(d_{ij}\right)_{\mathbf{ag}} = \mathrm{Diag}[(1-\mathbf{r_{ag}})] + \left(\frac{\mathbf{r_{ag}} \times \mathbf{s_{ag}} \times \mathbf{Q}}{\mathbf{s_{ag}} \cdot \mathbf{Q}} \otimes \vec{\mathbf{1}} \right), \text{ for } a \in \{\mathrm{v}, \mathrm{f}\}, \text{ and} \tag{M8}$$

$$\mathbf{F_g} = \begin{pmatrix} F_{1g} & 0 & 0 \\ 0 & F_{2g} & 0 \\ 0 & 0 & F_{3g} \end{pmatrix}.$$

Equation (M7) is derived by noting that $\mathbf{X}(t)$ is the number of adult females in each field, $\mathbf{D_{vg}}(\mathbf{X}(t))$ is the number of virgin females in each field following their dispersal, and $\mathbf{D_{fg}}\mathbf{D_{vg}}(\mathbf{X}(t))$ is the number of mated females (and hence eggs) in each field after their dispersal. $\mathbf{F_g}$ is the respective fecundities times DI larval survival in each field, and includes both male and female larvae. $\mathbf{W}_{RR}$, $\mathbf{W}_{RS}$, and $\mathbf{W}_{SS}$ give the relative numbers of each of the different genotypes following mortality due to *Bt* toxins, so their sum is the proportion of larvae surviving selection. $\mathbf{D_{ag}}$ is identical to $\mathbf{Z_{ag}}$ (equation M2) with $\mathbf{X}$ replaced by the vector of 1's, denoted $\vec{\mathbf{1}}$.

To model density-dependent (DD) survival, let $f[\mathbf{X}]$ be the function giving density-dependent larval survival following selection by *Bt* toxins:

$$f_g[\mathbf{X}] = f_g\begin{bmatrix} x_1 \\ x_2 \\ x_3 \end{bmatrix} = \begin{pmatrix} x_1 f_{1g}[x_1] \\ x_2 f_{2g}[x_2] \\ x_3 f_{3g}[x_3] \end{pmatrix} = \begin{pmatrix} x_1(1 + a_g x_1 / Q_1)^{-b_g} \\ x_2(1 + a_g x_2 / Q_2)^{-b_g} \\ x_3(1 + a_g x_3 / Q_3)^{-b_g} \end{pmatrix} \qquad \text{(M9)}$$

Note that the function $f_g[\mathbf{X}]$ depends on the densities of larvae within fields, and the number of larvae within *Bt* and refuge fields is converted into density per m^2 by dividing by the relative areas of *Bt* and refuge fields. Strength of density dependence may be different for the two generations. Female population sizes are given by the recursion equation

$$\mathbf{X}(t+1) = SR\, f_g[\mathbf{X}'(t)], \qquad \text{(M10)}$$

where *SR* is the female sex ratio, and the recursion recovers female densities after density dependence. Together, equations (M5) and (M10) give a full generation cycle for the resistance allele frequency and population density.

Sesamia nonagrioides has two complete generations a year, so the evolutionary model repeats equations M1-M10 allowing all parameter values to differ for the second generation. The second generation larvae (equation M10) may emerge as a third generation or enter diapause. The third generation may complete development to enter diapause in some years. Diapausing larvae from the second and third generation undergo strong density-dependent (DD) mortality before the winter.

Although the rate of diapause induction may be genotype-dependent, if, for example, *RR* genotypes develop more slowly than *SS* genotypes, we do not include this effect in our model, because the difference in development may be only a few days and the diapause induction window is about two weeks. To allow for DI overwintering mortality, larvae entering the winter survive with a survival rate of s_w, which is assumed to be independent of genotype and field.

3. Parameterization of Model

We used the best information available on the ecology of *S. nonagrioides* to estimate the parameters in the resistance evolution model. We refer to this set of parameter values as the "best parameter values" (BPV) and use model simulations based on these as the standard of comparison for the simulation experiments we conducted.

3.1. Initial resistant allele frequency (p_0)

The initial resistance allele frequency in *S. nonagrioides* was estimated by using an F_2 screen and a Bayesian estimator [25]. The expected frequency of resistance from a population in northeast Spain was 2.9×10^{-3}, with a 95% credibility interval of 0 to 0.0086. This was probably higher than the actual value in the population because the study was limited to 85 F_2 lines. *Sesamia nonagrioides* is distributed in the southern Paleartic from Spain and other Mediterranean countries through Turkey and east through Iran. Studies on geographical variation in the species reveal no evidence of any geographic barriers to gene flow, although the France-Spain populations show a hint of genetic differentiation from the eastern populations [26]. These results suggest that *S. nonagrioides* may be a single panmictic unit across its entire geographic range, and we can pool the results for Spain and Greece [25], resulting in an expected initial resistance allele frequency of 1.5×10^{-3}. Even this value may be higher than the actual value in the northeast Spain population because only 160 F_2 lines were screened, and no resistance alleles were recovered. In comparison, for *Ostrinia nubilalis*, 1184 F_2 lines were screened [27]. Hence we use both 2.9×10^{-3} and 1.5×10^{-3} as initial resistance allele frequencies in our simulations, and we assume that the latter is the best available estimate.

3.2. Adult movement (r_{iag} and s_{iag}) and assortative mating (α_g)

First flight. The first flight comprises adults emerging from overwintered individuals. Field observations during this flight indicated that >94% of females were mated when collected in light traps [28]. This result suggests either that virgin females are not attracted to light traps or that females mate before moving. Because second flight virgin females are caught at light traps at a higher rate (see below), the second hypothesis is more likely. In this case, males must search out sedentary virgin females, so male movement should be extensive. Mated female movement might also be extensive, because female movement to colonize maize fields early in the season has been reported [29,30]. There are no studies suggesting differential movement from *Bt* and/or non-*Bt* fields, differential attraction to either *Bt* or non-*Bt* fields, or local random/ nonrandom mating structure. Parameter values consistent with these data and considerations are given in Table B.

Table B. Parameterization of movement and mating for the first, second, and third flights of *S. nonagrioides*; *g* is for generation 1 - 3; *a* is for adult type, *v* = virgin female, *f* = mated female, *m* = male; *i* is for field type; α is the measure of local non-random mating; *r* is the proportion of adults leaving the field they are in; and *s* is the preference for a field type.

Generation	Adult Type	α_g	r_{iag}	s_{iag}
g = 1	*a* = *v*	0	0.05 for all *i*	1/3 for all *i*
	a = *f*		1.0 for all *i*	1/3 for all *i*
	a = *m*		1.0 for all *i*	1/3 for all *i*
g = 2	*a* = *v*	0	0.02 for all *i*	1/3 for all *i*
	a = *f*		0.04 for all *i*	1/3 for all *i*
	a = *m*		1.0 for all *i*	1/3 for all *i*
g = 3	*a* = *v*	0	0.02 for all *i*	See Table C
	a = *f*		1.0 for all *i*	See Table C
	a = *m*		1.0 for all *i*	See Table C

Second flight. Initial indications from laboratory observations of mating behavior suggested that females, unlike males, did not move before mating and mated mostly in the first scotophase after emergence [31]. Field observations indicated that 16 times more males than females were caught in light traps at a border between *Bt* (Event 176) and non-*Bt* maize [29]. Rubidium-marked second flight males were caught at pheromone traps with an equal frequency 0, 100, 200, 300, and 400 m from the release site, which suggests that males readily disperse 400 m to mate [32]. Pheromone

trap catches of *S. nonagrioides* males were uniform in *Bt* and non-*Bt* maize during the second flight, which indicates homogeneous mixing of males on a spatial scale of at least 200 m [29]. Two additional years of study confirmed these results for males of the second flight [33]. Thus, most males are able to leave their natal field, and, therefore, there is probably random mating between males and females within a radius of 400 m and beyond for some unknown distance [33]. If all males leave their natal field, using previous results [29], perhaps only 6% of females disperse. Of the females caught in the light traps, 68% were mated [29]. Thus, about 4% of mated females and 2% of virgin females might disperse. There are no data that indicate any movement preferences. Possible parameter values are given in Table B.

Third flight. No information is available for the third flight. Because the conditions are similar to the second flight, we assume that most of the parameters are similar to those of the second flight. However, mated female movement must be higher because *Bt* maize is mature and less attractive and females must find fields of green maize for oviposition. In addition, the proportion of *Bt* maize available will differ, because most of the maize is drying, unattractive and harvested before *S. nonagrioides* can complete a third generation. We assume that 1% of this long-season, early-planted maize is available for the third generation. Late season maize is primarily a short-season maize, sown as a second crop after barley, beans and peas, and forage maize. Before 2006, about 5% of the total annual maize area in northeast Spain was late season maize and after 2007, about 22% was late season maize (Table C). During 2006-2007, we assume that there was 15% late season maize. Forage maize is a late season maize and accounts for only 1% of the total maize area in northeast Spain and contributes to the refuge during the third generation.

The proportion of *Bt* maize in late season maize has increased rapidly (Table C). Before 2003, all of the short-season maize was non-Bt, because the only *Bt* maize varieties available were long-season varieties. After 2011, about 75% was a MON 810 short season variety. During 2004-2007 there was an intermediate situation, as indicated in Table C. Thus, for the third generation there has been a different proportion of *Bt* and non-*Bt* maize available than there was for the first two generations.

To model the difference in the proportion of *Bt* maize available for the 3rd generation, we could modify *Q* (the proportion of *Bt* maize available to the first two generations). Alternatively, because *s* and *Q* enter the movement equations in exactly the same way, we adjusted the third generation preference parameters, $s_{i\alpha 3}$, to adjust the *Bt* and non-*Bt* maize proportions available for virgin and mated females and males. Specifically the $s_{i\alpha 3}$ are adjusted as in Table C so that the %*Bt* and % refuge is correct for the third generation. All adult stages (virgin females, mated females and males) are assumed to have the same response. During the period before 2003, this is equivalent to 1% *Bt*

maize (Event 176) for the third generation. For the period after 2011, this is equivalent to 75% *Bt* maize (MON 810) for the third generation. Movement parameter values for r_{ia3} are given in Table B.

Table C. Percent *Bt* and refuge maize during the third generation of *S. nonagrioides*.

Ebro Valley	1998-2003 (1-5)[c]	2003-2005 (6-8)	2006-2007[a] (9-10)	2008-2011[b] (11-14)	2012- 2013 (15-16)
% late season maize/total maize	5	5	15	22	22
% late season MON 810 *Bt* maize/total late season maize (Q_{2l})	0	2.5	25	48	75
% Event 176 *Bt* maize/total late season maize (Q_{1l})	1	1	0	0	0
% forage maize/ total late season maize[d]	20	20	6.7	4.5	4.5
% non-Bt maize/ total late season maize	99	96.5	75	52	25
s_{1a3}	$Q_{1l}/3Q_1$	$Q_{1l}/3Q_1$	0	0	0
s_{2a3}	0	$Q_{2l}/3Q_2$	$Q_{2l}/3Q_2$	$Q_{2l}/3Q_2$	$Q_{2l}/3Q_2$
s_{3a3}	$(1-(s_{1a3}+s_{2a3})/3Q_3$	$(1-(s_{1a3}+s_{2a3})/3Q_3$	$(1-(s_{1a3}+s_{2a3})/3Q_3$	$(1-(s_{1a3}+s_{2a3})/3Q_3$	$(1-(s_{1a3}+s_{2a3})/3Q_3$

[a] An agronomically adequate short-season *Bt* variety (DKC5784YG) was commercialized in 2006
[b] An agronomically very good short-season *Bt* variety (DKC5277YG) was commercialized in 2010
[c] In brackets number of years of *Bt* maize cultivation in northeast Spain.
[d] Calculated by dividing 1% forage maize/ total maize in northeast Spain by % late season maize/ total maize (line 1 of this table).

3.3. Selection (k_{ig}, L_{ig}, h_{ig})

We assume that a single resistance allele gives *S. nonagrioides* resistance to both Event 176 and MON 810. Because Event 176 is not high-dose for the second and third generations, it is possible that during these generations Event 176 will select for weaker resistance alleles that would not give resistance to MON 810. However, the first generation selection by Event 176 would select against these weaker alleles and would select for resistance alleles that could confer resistance for MON 810.

We also assume that the offspring from the first and second flight will be exposed to *Bt* maize in proportion to the area planted. Selection will be different in Event 176 and MON 810, because toxin concentration in Event 176 declines after maize anthesis [34]. We compiled Event 176 toxin concentration data for different maize development stages (Table D) from previous studies [34-36]. The first study [34] clearly showed a decline in Cry1Ab concentration after anthesis, and the results from the second study [35] were consistent with these data [34]. Data presented by the third study [36] were not consistent with a decline in Cry1Ab concentration after anthesis. Data on the toxicity of Event 176 [37] provided strong evidence that Cry1Ab concentrations decline after anthesis. They showed that second generation *Ostrinia nubilalis* survived on Event 176, while first generation did not. In this study [37], the second generation was infested at anthesis and four weeks later had about half the survival as those infested on non-Bt plants. Because *O. nubilalis* is very sensitive to Cry1Ab, their results [37] showed that the concentration of Cry1Ab toxin declined after anthesis in Event 176. There is some survival of second generation *S. nonagrioides* on Event 176 [18,29], although the survivors may suffer from some sublethal effects from the low exposure to Cry1Ab toxin [33, 38]. Hence selection will differ on the first and second generations of *S. nonagrioides* for Event 176. By the time of the third generation, Event 176 will express even less Cry1Ab toxin, so the selection pressure will be even less.

Table D. Estimated concentration of Cry1Ab in Event 176 (μg/g FW)

	Fearing et al. [34]		Dutton et al. [35]		Obrist et al. [36]	
	mean	SE	mean	SE	mean	SE
Seedling	0.7	0.1				
Leaf 5			3.4	na		
Anthesis	2.44	0.31			1.2	0.15
Leaves at seed maturity	0.45	0			1.8	0.15
Senescing leaves	0.15	0.05				

Selection on MON 810 *Bt* maize will be similar in the first and second generation because larvae have not been observed to survive in either generation. We assume that MON 810 continues to express during the third generation, so selection is maintained for all three generations. Consequently, we assume selection parameters equivalent to a high dose for the first generation on Event 176 and all generations on MON 810 (Table E). The parameter values for second and third

generations on Event 176 allows for some selection against heterozygotes (similar to but weaker than the high dose strategy) and for susceptible survival to be 30% that on non-*Bt* plants. This encapsulates the assumption that during the second generation Event 176 weakly selects against heterozygotes (i.e., it does not simply favor the evolution of resistance), while the third generation is weaker still.

The parameter values used assume no cost of resistance. While there is a cost of resistance in most cases of *Bt* resistance [39], it is not possible to predict the cost. Because the value of the cost has a major impact on resistance evolution, in absence of evidence, the no cost assumption is a worst case analysis.

Table E. Survival rate for resistant (L_{ig}) and susceptible (k_{ig}) homozygotes and heterozygosity of resistance (h_{ig}) in the three patch types.

Patch Type	Generation	L_{ig}	k_{ig}	h_{ig}
i = 1, Event 176	g = 1	1.0	0.0001	0.001
	g = 2	1.0	0.3	0.3
	g = 3	1.0	0.7	0.5
i = 2, MON 810	g = 1	1.0	0.0001	0.001
	g = 2	1.0	0.0001	0.001
	g = 3	1.0	0.0001	0.001
i = 3, non-Bt	g = 1	1.0	1.0	0.5
	g = 2	1.0	1.0	0.5
	g = 3	1.0	1.0	0.5

3.4. Fecundity and DI survival (F_{ig})

Fecundity and density-independent mortality are combined in our model. Fecundity is different for females in the three flights. It was found that photoperiod experienced by larvae and adults affected fecundity [40]. Under conditions simulating the first flight (short days for larvae, long days for adults) average fecundity was 513 eggs/ female, and under conditions simulating the second flight (long days for larvae, long days for adults) average fecundity was 372 eggs/ female. Under conditions simulating the third flight, average fecundity was only 218 eggs/ female. We have estimated that density-independent egg and larval mortality is about 85-90%, which results in F_{i1} = 51.3-77.0 eggs/ female and F_{i2} = 37.2-55.8 eggs/ female. For the third generation, the major density-

independent mortality factor is the end of the growing season (harvesting and lack of heat) before the larvae reach a stage suitable for diapause.

3.5. Diapause and DI mortality in the third generation

Diapause is induced in first and second instars by the short day length during the last two weeks of August and occurs in the last instar [8]. If the second flight is late, a greater proportion of the second generation larvae enter diapause, and if the flight is earlier, a smaller proportion of the second generation larvae enter diapause. The quantitative relationship between the timing of the second flight and the proportion of larvae entering diapause is not known. Diapause is partially determined genetically [41, 42], so there is always a third flight in northeast Spain [12]. Consequently, we assume a simple linear relationship between the time of the second flight and the proportion of offspring entering diapause, which is consistent with these observations (Fig E).

Fig E. Relationship between time of the second flight of *S. nonagrioides* (50% of flight) and proportion of second generation larvae entering diapause.

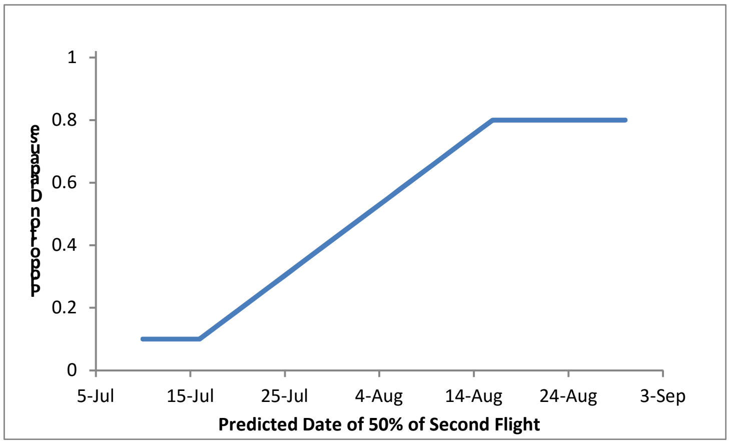

DI mortality in the third generation will vary from year to year because some years are too cold for the third generation to complete development, and some years are warm enough to allow the third generation to complete development to diapausing mature larvae. We collected the historical temperature records for three sites in northeast Spain (Tauste (Zaragoza), Montañana (Zaragoza), and Candasnos (Huesca)) and using the development model for *S. nonagrioides* in Figure 2e and Tables 2 and 7 in López et al. [12], we calculated the degree-days (^{o}d) remaining in a year for the third generation to complete development, and converted these values into mortality rates

(proportion that could not complete development). Specifically, we used post-diapause development to predict the ^{o}d to 50% of the first flight based on Figure 2e in López et al. [12], constraining the prediction so that extrapolations of the equation always occurred after 30 April. To this we added 730 ^{o}d and 491 ^{o}d, which according to Table 7 in López et al. [12] would give the total predicted ^{o}d to the middle of the third flight. From the weather data, we calculated the total ^{o}d in the year until 31 Oct (typically the latest harvest date) and subtracted the previous number to estimate the ^{o}d remaining in a year for the third generation to complete development. In any year, the values for the three sites were similar and these were averaged. Using these annual averages, we assumed that the third generation required 434 ^{o}d to develop to the final instar as in Table 2 in López et al. [12], that 10 ^{o}d accumulate each day during the latter part of the third larval generation, and that the distribution of the third flight lasted 28 days with a peak in the middle. If the middle of the third flight occurred with 434 ^{o}d left in the year, then we assumed that the midpoint of the flight was a cutoff date, and larvae from the first half of the third flight had sufficient heat to mature and diapause, but larvae from the second half the flight did not have sufficient heat and would die. If there was more or less than 434 ^{o}d left, we calculated how the cutoff date would change by subtracting 434 and dividing by 10 to calculate the number of days before or after the peak that the cutoff date should be adjusted, and used this cutoff date to determine the area under the third flight distribution with offspring that had insufficient heat to complete development and be assumed to die.

We found that the third generation is predicted to be successfully completed in two of ten years in northeast Spain (Table F). We also noted that there are three types of years predicted for the third generation: a) low diapause and low mortality (2006, 2011); b) low diapause and high mortality (2005, 2012); and high diapause and high mortality (the rest of the years). We used these values in our simulation model and randomly selected one of the years for use in the model before 2004 and after 2013. The mortality rates were multiplied by 218 eggs/ female to provide estimates of fecundity for the third generation in the model.

Table F. Predicted DI mortality rates for the third generation due to not completing development to diapause, and proportion of larvae diapausing in the second generation.

Year	Mortality Rate	Proportion Diapause
2004	1	0.8
2005	0.9461	0.2129
2006	0.1648	0.1
2007	1	0.7548
2008	1	0.6871
2009	1	0.8
2010	1	0.8
2011	0.4936	0.3032
2012	0.9962	0.4839
2013	1	0.8

3.6. Density dependent (a_g, b_g) and overwinter survival (s_w)

First generation. There is weaker density-dependent larval mortality (during the late instars) during the first generation, compared to that in the second generation diapausing larvae. In addition, final instar population density in the first generation is typically less than that in the second generation. A few studies have estimated survival of larvae during the first generation (Fig F), mostly in the greenhouse on whole plants with some experiments in the field (unpublished results). It is unlikely that there is overcompensating density-dependence in the species, so we used the constraint $b \leq 1.0$. Larval survival was higher on greenhouse plants than in the field, but the final larval densities on the greenhouse plants (approximately 4 per plant) are not higher than the highest observed field densities. The greenhouse plant model probably is the upper value for possible survival rates in the field. We used the parameters estimated from the field data in the model. Although there are several combinations of a_1 and b_1 that would fit these field data adequately, we used $a_1 = 0.1$ and $b_1 = 0.75$; the parameterized model is shown in Fig F.

Fig F. Model and data for density-dependent survival of first generation larval *S. nonagrioides*. Symbols: dark green triangles are whole plant trials in greenhouse; orange squares are field trials. Model fit to data: green line for greenhouse and orange line for field. Green: a = 0.02, b = 1.0; Orange: a = 0.1, b = 0.75.

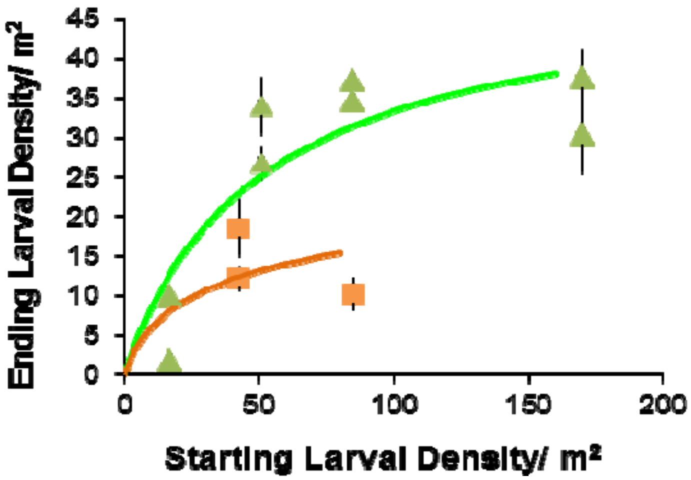

Second generation. Some of the second generation larvae will emerge to start a third generation, but these will have a low probability of reproduction compared to those that enter diapause. Those that enter diapause will compete to inhabit the maize root crown. Because above-ground maize residue is typically cut into pieces after harvest, and the residue is tilled into the soil, larval survival tends to be limited to about one individual per stalk for the overwintering generation [43]. This individual survives these disturbances in the stiff maize root crown. Under these assumptions, a_2 = 0.11 and b_2 = 0.999 results in a maximum of about 1 larva/stalk surviving the winter, assuming a plant density of about 85,000 plants/ha (Fig G).

Fig G. Model for density-dependent survival of diapausing second generation larval *S. nonagrioides*.

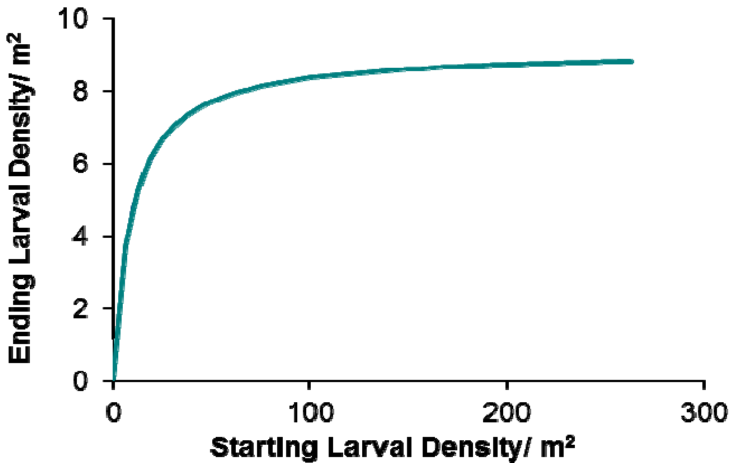

Third generation. Density-dependent survival of the third generation is treated as identical to the second generation, because both generations are overwintering as diapausing larvae. Because these larvae are usually on different plants than the second generation larvae, their survival is calculated separately from that of the second generation. The number of second and third generation diapausing larvae are added together before overwintering mortality.

Overwintering DI mortality occurs after this fall DD mortality. Overwintering mortality factors are primarily abiotic [43], probably related to cold temperatures. It was reported about 5% overwintering survival in southwestern France, but this included aboveground larvae on unharvested plants; none of these larvae survived overwintering [43]. Moreover, when considering these larvae, they found that about half of the plants had more than one larva per plant. Hence, the density-dependent function described above takes into account mortality of these larvae. They also found that overwintering survivors were solely in the root crown. Because we have already accounted for mortality in the maize stems in second and third generation, we calculated overwinter survival of larvae in root crowns from Figure 5A in Gillyboeuf et al. [43]. Root crown infestation rate was 0.542 at the beginning of November and 0.057 at the beginning of April, giving an overwintering survival rate of 10.4%.

3.7. Field rotation in the next year

The proportion of maize area that was cultivated with maize in two consecutive years is given in Table G. In northeast Spain about 55% of the area has maize for two consecutive years and 45% is rotated to another crop. These rates are considerably more variable in other maize cultivation regions of Spain. There are no data related to rotation of *Bt* maize and non-*Bt* refuge maize, but if Spanish farmers behave similar to US farmers, they will tend not to rotate between *Bt* and non-*Bt* maize on the same field. US farmers tend to plant the more expensive *Bt* maize on their best land, so that they can gain the maximum benefit and suffer the smallest losses in the refuge maize.

Table G. Percent of maize area that is cultivated to maize in two consecutive years.

Region of Spain	Comunidad Autónoma	2007-2008	2008-2009	2009-2010
Northeast	Cataluña	50.6	59.0	58.3
	Aragón	51.9	55.3	50.6
Central	Madrid	92.6	82.5	64.0
	Castilla-La Mancha	43.1	44.4	53.4
Southwest	Extremadura	77.1	57.6	63.5
	Andalucía	43.1	29.6	32.9

Source: Encuesta sobre Superficies y Rendimientos Cultivos (ESYRCE). Ministerio de Agricultura, Alimentación y Medio Ambiente http://www.marm.es/es/estadistica/temas/encuesta-sobre-superficies-y-rendimientos-de-cultivos-esyrce-/

3.8. Refuge compliance

Farmers are required by the seed provider to plant 20% of their maize as a non-*Bt* refuge when the *Bt* maize surface is ≥ 5 ha. The compliance with this requirement in the Ebro Valley (Huesca, Lleida and Zaragoza) has increased from 64% in 2006 to 93% in 2011 (Table H). It was likely lower than 64% prior to 2006.

Table H. Percent of farmers that planted the required conventional maize refuge on their farm.

Year	Compliance (%)[a]
2006	64
2007	74
2008	82
2009	81
2010	88
2011	93

[a] Farmer surveys with farm questionnaires sponsored by ANTAMA (Spanish Foundation supporting the use of new technologies in agriculture) addressing the implementation of non-Bt refuges by 100 Spanish farmers (from northeast Spain) who cultivated maize MON 810 (data 2006-2008 from ANTAMA, data 2009-2011 from EFSA [44]).

3.9. Natural enemy mortality

Natural enemies are assumed to be insignificant in *S. nonagrioides* population dynamics. Insect diseases have not been studied for this species, and to our knowledge what little information that exists does not identify any specific entomopathogenic agents [43, 45], and parasitism rates have been low in Spain (Table I) and elsewhere [45, 46]. Because females oviposit inside maize leaf sheaths and larvae immediately bore into the maize stem, predation rates are likely to be low.

Table I. Parasitoids in overwintering *Sesamia nonagrioides* (unpublished data)

Region in Spain	*Sesamia* larvae[a]	Tachinids	% parasitized
Northeast	3737	38	1.017
Central	2840	8	0.282
Southwest	2531	6	0.237
Total	9108	52	0.571

[a] Overwintering last instar larvae of *S. nonagrioides* were collected from maize fields, transferred to the laboratory and kept at diapausing conditions. After 2-3 months, larvae were placed in conditions to break diapause. The incidence of parasitoids was checked every week during diapause and every day after being placed in conditions to break diapause.

4. Model Experiments

4.1. Initial R *allele frequency*

Many researchers have shown that the initial *R* allele frequency is an important determinant of the time to resistance failures. As indicated above, the *R* allele frequency has been estimated using F_2 screens [25]. These estimates, however, may have overestimated the *R* allele frequency because of the limited number of F_2 lines screened. Hence, we conducted two simulations with different initial *R* allele frequencies: 2.9×10^{-3}, which corresponds to the measured expected value from Spain, and 1.5 x 10-3, which corresponds to the measured expected for Spain and Greece combined.

4.2. Female dispersal

It has been shown that higher rates of female dispersal increased the rate of resistance evolution [22]. As noted above, movement of female *S. nonagrioides* is believed to be restricted for first generation unmated females, second generation unmated and mated females, and third generation unmated females, with less than 5% of females leaving their natal fields. To investigate how increased female movement could affect the rate of resistance evolution, we increased female movement in all generations to the maximum possible of 1.0 (all females leave their natal habitat) and compared the result to the BPV simulations with an initial allele frequency = 0.0015.

4.3. Adoption rate

As indicated in Figure 2 in the main text, the initial adoption rate of *Bt* maize was low, and it has only been in the past five years that adoption has reached appreciable levels. When adoption is low, selection for resistance will be low. Hence it is possible that the durability of *Bt* maize in northeast Spain is related to the initial low adoption rate. While it is widely agreed that low adoption will reduce the risk of resistance evolution, we quantified this by comparing the observed initial low adoption rates with hypothetical high adoption rates. We assumed that Event 176 and MON 810 were adopted at their observed relative proportions, but totalling 80% of the maize area from the first year. We also considered if either Event 176 or MON 810 by themselves had been adopted at 80% of the maize area from the first year onward, and compared the rate of resistance evolution to the BPV simulations with an initial allele frequency = 0.0015.

4.4. Spatial structure of refuge

As indicated above, farm sizes in northeast Spain averaged 6.3 – 9.0 ha. This results in a finely divided landscape with *Bt* and non-*Bt* maize in close proximity. Hence, even though compliance to planting 20% non-*Bt* maize refuge was initially low, there were many non-*Bt* maize fields nearby the *Bt* fields during the early years of adoption, and these functioned as unplanned, happenstance refuges. As adoption increased, compliance also increased, so the initial low compliance (Table H) has not created a concern about insufficient refuge or poor refuge placement.

We investigated what could have happened in northeast Spain had refuge been absent or too far away. In this case, refuge and *Bt* fields would have been aggregated into large patches of refuge and *Bt* maize, and the main effect on the population dynamics would have been to generate non-random positive assortative mating. The assortative mating would be associated with the *Bt* crop once the *Bt* crop predominated in the landscape, but it would be weak in the non-*Bt* refuge, because the refuge is small and the perimeters bordering with *Bt* corn are large compared to the area of the refuge. Consequently, proportionately fewer adults from the refuge would stay in the refuge than adults from the *Bt* corn, and assortative mating would be higher in *Bt* fields than in the refuge.

To model this, we assumed that there was no assortative mating until the refuge dropped below 50%. Then assortative mating occurred only in the *Bt* crops. We varied positive assortative mating in the *Bt* fields from 0 to 0.04 (4% assortative mating) for all three generations.

4.5. Interactions among the factors

Interactions between initial *R* allele frequency and female movement, female movement and assortative mating, and assortative mating and adoption rate were also examined by varying bother factors independently.

4.6. External colonization

We modelled colonization by first determining the equilibrium population of *S. nonagrioides* in northeastern Spain when there is no *Bt* maize planted. We then varied the number of colonists as a proportion of this equilibrium population. Quantitatively, we considered migration rates from 0 to 4.5% of the equilibrium population, a very large range of possibilities. We assumed that all colonists contributed larvae to the first generation at the same rate as residents, and that colonists were all

homozygous susceptible for resistance (*SS* genotypes). Thus the effect of external colonization was to increase the size of the first generation larval population, and not to mate with the resident population.

5. References

1. Cordero A, et al. (1998) Population dynamics and life-cycle of corn borer in south atlantic european coast. *Maydica* 43: 5-12.

2. Anglade P (1972) Les Sesamie. In Entomologie Appliquée á l'Agriculture. Tomme II. Lépidoptères (Deuxiemme Volumen), A. S. Balachowsky, Ed. (Masson et Cie, Paris), pp. 1389-1401.

3. Alfaro A (1972) Notas sobre *Ostrinia nubilalis* (Hub.) y *Sesamia nonagrioides* (Lef.). *An INIA Ser Prot Veg* 2 : 145-170.

4. Galichet PF (1982) Hibernation d¨une populationde *Sesamia nonagrioides* Lef. (Lep: Noctuidae) en France méridionale. *Agronomie* 2 : 561-566.

5. Eizaguirre M, et al. (2002) Maize phenology influences field diapause induction of *Sesamia nonagrioides* (Lepidoptera: Noctuidae). *Bull Entomol Res* 92 : 439–443.

6. Hilal A (1977) Mise en evidence d'un etat de diapause vraie chez *Sesamia nonagrioides* Lef. (Lepidoptera Noctuidae). *C R Acad Sci Paris* 286: 365-367.

7. Figueiredo D, Araújo J (1990) Introduçao à Protecçao Integrada da Cultura do Milho de regadio. *Bol San Veg Plagas* 16: 135-138.

8. Eizaguirre M, Albajes R (1992) Diapause induction in the stem corn borer, *Sesamia nonagrioides* (Lepidoptera: Noctuidae). *Entomol Gen* 17: 277-283

9. Eizaguirre M, et al. (1994) Thermoperiodism, photoperiodism, and sensitive stage of *Sesamia nonagrioides* (Lepidoptera: Noctuidae). *J Insect Physiol* 40: 113-119.

10. Fantinou AA, et al. (1995) Diapause induction in the *Sesamia nonagrioides* (Lepidoptera: Noctuidae) effect of photoperiod and temperature. *Environ Entomol* 2: 1458-1466.

11. Gadenne C, et al. (1997) Occurrence of non-stationary larval moult during diapauses in the corn-stalk borer, *Sesamia nonagrioides* (Lepidoptera: Noctuidae). *J Insect Physiol* 43: 425-431.

12. López C, et al. (2001) Phenological model for *Sesamia nonagrioides* (Lepidoptera: Noctuidae). *Environ Entomol* 30: 23-30.

13. Brookes G (2007) The benefits of adopting genetically modified, insect resistant (Bt) maize in the European Union (EU): First results from 1998-2006 plantings. (PG Economics Ltd.).

14. Gómez-Barbero M, et al. (2008) Bt corn in Spain-the performance of the EU´s first GM crop. *Nat. Biotechnol* 26: 384-386

15. Gómez-Barbero M, et al. (2008) Adoption and performance of the first GM crop introduced in EU agriculture: Bt maize in Spain. (Joint Research Centre, Institute for Prospective Technological Studies, Scientific and Technical Reports).

16. Censo Agrario (2009) Instituto Nacional de Estadística. http://www.ine.es/jaxi/menu.do?type=pcaxis&path=%2Ft01%2Fp042/E01&file=inebase&L=0.

17. Messeguer J, et al. (2006) Pollen-mediated gene flow in maize in real situations of coexistence. *Plant Biotechnol J* 4: 633-645.

18. Farinós GP, et al. (2004) Resistance monitoring of field populations of the corn borers *Sesamia nonagrioides* and *Ostrinia nubilalis* after 5 years of Bt maize cultivation in Spain. *Entomol Exp Appl* 110: 23-30.

19. Farinós GP, et al. (2011) Comparative assessment of the field-susceptibility of *Sesamia nonagrioides* to the Cry1Ab toxin in areas with different adoption rates of Bt maize and in Bt-free areas. *Crop Prot* 30: 902-906.

20. EFSA. Scientific Opinion on the annual post-market environmental monitoring (PMEM) report from Monsanto Europe S.A. on the cultivation of genetically modified maize MON 810 in 2013. EFSA Journal 2015. 13(3): 4039.

21. Comins HN (1977) The development of insecticide resistance in the presence of migration. *J Theor Biol* 64: 177-197

22. Ives AR, Andow DA (2002) Evolution of resistance to *Bt* crops: Directional selection in structured environments. *Ecology Letters* 5: 792-801

23. Alstad DN, Andow DA (1995) Managing the evolution of insect resistance to transgenic plants. *Science* 268: 1894-1896.

24. Andow DA, Ives AR (2002) Monitoring and adaptive resistance management. *Ecol Appl* 12: 1378–1390.

25. Andreadis SS, et al. (2007) Frequency of resistance to *Bacillus thuringiensis* toxin Cry1Ab in Greek and Spanish population of *Sesamia nonagrioides* (Lepidoptera: Noctuidae). *J Econ Entomol* 100: 195-201.

26. De la Poza M, et al. (2008) Bt maize resistance management: genetic structure of *Sesamia nonagrioides* (Lefebvre) populations in the Mediterranean area. *Environ Entomol* 37: 1354-1360.

27. Bourguet D, et al. (2003) Frequency of alleles conferring resistance to *Bt* maize in French and US corn belt populations of the European corn borer, *Ostrinia nubilalis*. *Theor Appl Genet* 106: 1225-1233.

28. López C, et al. (1999) Influencia de la planta de maíz en el apareamiento de *Sesamia nonagrioides* Lefèbvre (Lepidoptera: Noctuidae). *Inv Agr: Prod Prot Veg* 14 : 415–422.

29. Eizaguirre M, et al. (2004) Dispersal capacity in the Mediterranean corn borer, *Sesamia nonagrioides* (Lepidoptera: Noctuidae). *Entomol Exp Appl* 113: 25-34.

30. Larue P (1984) La Sésamie du maïs (*Sesamia nonagrioides* Lef.). Dégâts et actualisation de lutte. *Défense Végétaux* 227: 163–181.

31. López C, et al. (2003) Courtship and mating behaviour of the Mediterranean corn borer, *Sesamia nonagrioides* (Lepidoptera: Noctuidae). *Spanish J Agric Res* 1 : 43–51.

32. Albajes R, et al. (2004) Testing rubidium marking for measuring adult dispersal of the corn borer *Sesamia nonagrioides*: first results. *IOBC/WPRS Bull* 27 (3): 15-22.

33. Eizaguirre M, et al. (2006) Six years after the commercial introduction of Bt maize in Spain: field evaluation, impact and future prospects. *Transg Res* 15: 1–12.

34. Fearing PL, et al. (1997) Quantitative analysis of CryIA(b) expression in Bt maize plants, tissues, and silage and stability of expression over successive generations. *Mol Breed* 3: 169–176.

35. Dutton A, et al. (2002) Uptake of Bt-toxin by herbivores feeding on transgenic maize and consequences for the predator *Chrysoperla carnea*. *Ecol Entomol* 27: 441-447.

36. Obrist LB, et al. (2006) Exposure of arthropod predators to Cry1Ab toxin in Bt maize fields. *Ecol Entomol* 31: 143–154.

37. Zoerb AC, et al. (2003) Larval distribution and survival of second generation European corn borer, *Ostrinia nubilalis* (Hübner) (Lepidoptera: Crambidae) on Event 176 Bt Corn. *Crop Prot* 22: 179–184.

38. Eizaguirre M, et al. (2005) Sublethal effects of *Bacillus thuringiensis* on larval development in *Sesamia nonagrioides J Econ Entomol* 98: 237-247.

39. Gasmann AJ, et al. (2009) Fitness costs of insect resistance to *Bacillus thuringiensis*. *Annu Rev Entomol* 54: 147–163.

40. Fantinou AA, et al. (2004) Reproductive responses to photoperiod and temperature by diapausing and nondiapausing populations of *Sesamia nonagrioides* Lef. (Lepidoptera – Noctuidae). *Physiol Entomol* 29: 169–175.

41. Eizaguirre M, et al. (1998) Juvenile hormone and diapause in the Mediterranean corn borer, *Sesamia nonagrioides*. *J Insect Physiol* 44: 419-425.

42. Eizaguirre M, et al. (2005) Relationship between an increase of juvenile hormone titer in early instars and the induction of diapause in fully grown larvae of *Sesamia nonagrioides*. *J Insect Physiol* 51: 1127-1134.

43. Gillyboeuf N, et al.(1994) Cold hardiness and overwintering strategy of the pink maize stalk borer, *Sesamia nonagrioides* Lef. (Lepidoptera, Noctuidae). *Oecologia* 99: 366-373.

44. EFSA (2013) Scientific Opinion on the annual Post-Market Environmental Monitoring (PMEM) report from Monsanto Europe S.A. on the cultivation of genetically modified maize MON 810 in 2011. *EFSA Journal* 11(12), 3500.

45. Figueiredo D, Araújo J (1996) Mortality factors of *Sesamia nonagrioides* Lef. (Lepidoptera: Noctuidae) in Portugal. I. Parasitoids. *Bol San Veg Plagas* 22: 251-260.

46. Galichet PF, et al. (1985) Bioecology of *Lydella thompsoni* Herting, [Dip. Tachinidae] within the Rhone Delta in Southern France. *Entomophaga* 30: 315-328.
