# Supplementary material for: Sixteen Years of Bt Maize in the EU Hotspot: Why Has Resistance Not Evolved?
Source: PLoS One. 2016 May 4;11(5):e0154200. doi: 10.1371/journal.pone.0154200 (PMC4856266; doi:10.1371/journal.pone.0154200)
Supplement: S2 File — (PDF) [file pone.0154200.s002.pdf]

| | | | |
|---|---|---|---|
| Southwest Spain | 1999 | Jarilla | N 40º 10' 5.26" W 6º 0' 15.69" |
| | 2000 | Montijo | N 38º 54' 57.47" W 6º 37' 23.97" |
| | 2002 | Tocina | N 37º 36' 17.94" W 5º 42' 48.92" |
| | | Los Palacios | N 38º 40' 30.11" W 6º 8' 28.2" |
| | | Badajoz | N 38º 56' 3.67" W 6º 54' 36.82" |
| | 2003 | Montijo | N 38º 54' 57.47" W 6º 37' 23.97" |
| | | Torremayor | N 38º 54' 29.79" W 6º 32' 38.78" |
| | 2004 | Azanaque | N 37º 35' 58.33" W 5º 35' 53.05" |
| | | San José de la Rinconada | N 37º 31' 10.48" W 5º 56' 41.33" |
| | 2004 | Veredón | N 37º 50' 45.13" W 4º 56' 43.4" |
| | | La Matilla | N 37º 22' 16.3" W 6º 21' 55.53" |
| | | Acedera | N 39º 3' 28.3" W 5º 33' 2.66" |
| | | Madrigalejo | N 39º 6' 20.4" W 5º 39' 25.13" |
| | 2005 | Guadiana del Caudillo | N 38º 57' 37.36" W 6º 41' 6.94" |
| | | El Torviscal | N 39º 5' 0.61" W 5º 42' 25.27" |
| | | Los Guadalperales | N 39º 6' 20.58" W 5º 39' 25.13" |
| | | Zurbarán | N 39º 4' 2.07" W 5º 40' 53.97" |
| | 2007 | Alcolea | N 37º 55' 43.8" W 4º 36' 41.09" |
| | | Peñaflor | N 37º 43' 43.93" W 5º 19' 40.55" |
| | | El Calonge | N 37º 39' 9.96" W 5º 24' 37.68" |
| | 2011 | La Ina | N 36º 38' 45.02" W 6º 2' 33.06" |
| | | José Antonio | N 36º 39' 39.55" W 5º 52' 27.61" |
| | | Riolobos | N 39º 55' 1.96" W 6º 17' 26.94" |
