# Supplementary material for: Correction: Sixteen Years of Bt Maize in the EU Hotspot: Why Has Resistance Not Evolved?
Source: PLoS One. 2016 Jul 25;11(7):e0160294. doi: 10.1371/journal.pone.0160294 (PMC4959677; doi:10.1371/journal.pone.0160294)
Supplement: S2 File — (PDF) [file pone.0160294.s001.pdf]

**Maize growing area for larvae collection**	**Year**	**Location**	**GPS coordinates**
Northeast Spain	1999	Lleida	N 41º 36' 55.82" E 0º 35´ 32.87"
		Cabañas de Ebro	N 41º 47' 35.68" W 1º 10´ 17.63"
	2000	Lleida	N 41º 36' 55.82" E 0º 35´ 32.87"
		Cabañas de Ebro	N 41º 47' 35.68" W 1º 10´ 17.63"
	2001	Lleida	N 41º 36' 55.82" E 0º 35´ 32.87"
		Cabañas de Ebro	N 41º 47'35.68" W 1º10´17.63"
		Biota	N 42º 15' 55.19" W 1º 11´53.49"
	2002	Lleida	N 41º 36' 55.82" E 0º 35´ 32.87"
		Cabañas de Ebro	N 41º 47' 35.68" W 1º 10´ 17.63"
		Quinto	N 41º 25' 48.68" W 0º 29´ 1.44"
		Villamayor	N 41º 41´ 4.85" W 0º 47´ 34.47"
	2003	Lleida	N 41º 36' 55.82" E 0º 35´ 32.87"
		Cabañas de Ebro	N 41º 47' 35.68" W 1º 10´ 17.63"
	2004	Zuera	N 41º 51' 22.11" W 0º 45´ 18.72"
		Almudévar	N 41º 57' 37.42" W 0º 44' 58.49"
		Bellvis	N 41º 39' 8.64" E 0º 50' 12.47"
		Huerto	N 41º 57' 26.02" W 0º 12' 13.9"
	2005	Mallén	N 41º 52' 59.05" W 1º 25' 53.05"
	2006	Tornabous	N 41º 40' 54.34" E 1º 3' 40.98"
		Zuera	N 41º 51' 22.11" W 0º 45´ 18.72"
		Candasnos	N 41º 29' 0.14" E 0º 5' 10.47"
		Bell-llos de Urgel	N 41º 35' 55.58" E 0º 47' 4.64"
	2008	Erla	N 42º 5' 46.17" W 1º 0' 13.09"
		Sta Anastasia	N 42º 8' 14.35" W 1º 16' 18.43"
		Gimenells	N 41º 39' 43.5" E 0º 24' 45.23"
		Candasnos	N 41º 29' 0.14" E 0º 5' 10.47"
	2009	Épila	N 41º 33' 52.84" W 1º 14' 55.74"
		Ejea de los Caballeros	N 42º 9´16.5" W 1º 11´ 4.09"
		Candasnos	N 41º 29' 0.14" E 0º 5' 10.47"
	2011	Alfamén	N 41º 26' 27.14" W 1º 19' 29.73"
		Agramonte	N 41º 50' 19.84" W 1º 46' 31.16"
		Valtierra	N 42º 13' 28.72" W 1º 38' 43.8"
		Alagón	N 41º 47' 19.99" W 1º 6' 19.39"
Central Spain	1999	Azuqueca de Henares	N 40º 35' 19.22" W 3º 15' 54.21"
		Estremera	N 40º 8' 0.96" W 3º 6' 5.17"
		Chinchilla	N 38º 54' 37.5" W 1º 43' 39.25"
	2000	Estremera	N 40º 8' 0.96" W 3º 6' 5.17"
		Sta. Cruz de la Zarza	N 38º 48' 23.89" W 2º 4' 48.82"
		Albacete	N 39º 3' 28.18" W 1º 53' 13.34"
	2001	Estremera	N 40º 8' 0.96" W 3º 6' 5.17"
		Albacete	N 39º 3' 28.18" W 1º 53' 13.34"
	2002	Estremera	N 40º 8' 0.96" W 3º 6'5.17"
	2003	Estremera	N 40º 8' 0.96" W 3º 6' 5.17"
		Villamanrique	N 38º 33' 11.14" W 3º 1' 0.6"
		Aranjuez	N 40º 2' 36.17" W 3º 37' 13.3"
	2005	Estremera	N 40º 8' 0.96" W 3º 6' 5.17"
	2006	Motilleja	N 39º 9' 39.65" W 1º 45' 7.37"
		Santa Ana	N 38º 53' 38.1" W 1º 59' 37.8"
	2008	Tarazona de la Mancha	N 39º 15' 18.3" W 1º 58' 48.41"
		Barrax	N 39º 01' 26.1" W 2º 10' 38.6"
		Aguas Nuevas	N 38º 54' 44.6" W 1º 55' 59.6"
	2010	Motilleja	N 39º 9' 39.65" W 1º 45' 7.37"
		La Herrera	N 38º 57'17.8" W 2º 07' 17.6"
		Aguas Nuevas	N 38º 54' 44.6" W 1º 55' 59.6"
Southwest Spain	1999	Jarilla	N 40º 10' 5.26" W 6º 0' 15.69"
	2000	Montijo	N 38º 54' 57.47" W 6º 37' 23.97"
	2002	Tocina	N 37º 36' 17.94" W 5º 42' 48.92"

		Los Palacios	N 38º 40' 30.11" W 6º 8' 28.2"
		Badajoz	N 38º 56' 3.67" W 6º 54' 36.82"
	2003	Montijo	N 38º 54' 57.47" W 6º 37' 23.97"
		Torremayor	N 38º 54' 29.79" W 6º 32' 38.78"
	2004	Azanaque	N 37º 35' 58.33" W 5º 35' 53.05"
		San José de la Rinconada	N 37º 31' 10.48" W 5º 56' 41.33"
	2004	Veredón	N 37º 50' 45.13" W 4º 56' 43.4"
		La Matilla	N 37º 22' 16.3" W 6º 21' 55.53"
		Acedera	N 39º 3' 28.3" W 5º 33' 2.66"
		Madrigalejo	N 39º 6' 20.4" W 5º 39' 25.13"
	2005	Guadiana del Caudillo	N 38º 57' 37.36" W 6º 41' 6.94"
		El Torviscal	N 39º 5' 0.61" W 5º 42' 25.27"
		Los Guadalperales	N 39º 6' 20.58" W 5º 39' 25.13"
		Zurbarán	N 39º 4' 2.07" W 5º 40' 53.97"
	2007	Alcolea	N 37º 55' 43.8" W 4º 36' 41.09"
		Peñaflor	N 37º 43' 43.93" W 5º 19' 40.55"
		El Calonge	N 37º 39' 9.96" W 5º 24' 37.68"
	2011	La Ina	N 36º 38' 45.02" W 6º 2' 33.06"
		José Antonio	N 36º 39' 39.55" W 5º 52' 27.61"
		Riolobos	N 39º 55' 1.96" W 6º 17' 26.94"
